# Very short sleep duration reveals a proteomic fingerprint that is selectively associated with incident diabetes mellitus but not with incident coronary heart disease: a cohort study

**DOI:** 10.1186/s12916-024-03392-1

**Published:** 2024-04-23

**Authors:** Thomas Svensson, Akiko Kishi Svensson, Mariusz Kitlinski, Gunnar Engström, Jan Nilsson, Marju Orho-Melander, Peter M. Nilsson, Olle Melander

**Affiliations:** 1 Department of Clinical Sciences, Lund University, Skåne University Hospital, CRC, Jan Waldenströms Gata 35, 20502 Malmö, Sweden; 2 Precision Health, Department of Bioengineering, Graduate School of Engineering, the University of Tokyo, 7-3-1 Hongo, Bunkyo-Ku, Tokyo, 113-8655 Japan; 3 Graduate School of Health Innovation, Kanagawa University of Human Services, Kawasaki-Ku, Kawasaki-Shi, Kanagawa, Japan; 4 Department of Diabetes and Metabolic Diseases, the University of Tokyo, 7-3-1 Hongo, Bunkyo-Ku, Tokyo, 113-0033 Japan; 5 Department of Cardiology, Skåne University Hospital, Malmö, Sweden; 6 Department of Internal Medicine, Skåne University Hospital, Malmö, Sweden

**Keywords:** Incident coronary heart disease, Incident diabetes, Inflammation, Lasso, Machine learning, Proteomic markers, Sleep duration

## Abstract

**Background:**

The molecular pathways linking short and long sleep duration with incident diabetes mellitus (iDM) and incident coronary heart disease (iCHD) are not known. We aimed to identify circulating protein patterns associated with sleep duration and test their impact on incident cardiometabolic disease.

**Methods:**

We assessed sleep duration and measured 78 plasma proteins among 3336 participants aged 46–68 years, free from DM and CHD at baseline, and identified cases of iDM and iCHD using national registers. Incident events occurring in the first 3 years of follow-up were excluded from analyses. Tenfold cross-fit partialing-out lasso logistic regression adjusted for age and sex was used to identify proteins that significantly predicted sleep duration quintiles when compared with the referent quintile 3 (Q3). Predictive proteins were weighted and combined into proteomic scores (PS) for sleep duration Q1, Q2, Q4, and Q5. Combinations of PS were included in a linear regression model to identify the best predictors of habitual sleep duration. Cox proportional hazards regression models with sleep duration quintiles and sleep-predictive PS as the main exposures were related to iDM and iCHD after adjustment for known covariates.

**Results:**

Sixteen unique proteomic markers, predominantly reflecting inflammation and apoptosis, predicted sleep duration quintiles. The combination of PSQ1 and PSQ5 best predicted sleep duration. Mean follow-up times for iDM (*n* = 522) and iCHD (*n* = 411) were 21.8 and 22.4 years, respectively. Compared with sleep duration Q3, all sleep duration quintiles were positively and significantly associated with iDM. Only sleep duration Q1 was positively and significantly associated with iCHD. Inclusion of PSQ1 and PSQ5 abrogated the association between sleep duration Q1 and iDM. Moreover, PSQ1 was significantly associated with iDM (HR = 1.27, 95% CI: 1.06–1.53). PSQ1 and PSQ5 were not associated with iCHD and did not markedly attenuate the association between sleep duration Q1 with iCHD.

**Conclusions:**

We here identify plasma proteomic fingerprints of sleep duration and suggest that PSQ1 could explain the association between very short sleep duration and incident DM.

**Supplementary Information:**

The online version contains supplementary material available at 10.1186/s12916-024-03392-1.

## Background

The associations between short or long sleep durations and diabetes mellitus (DM) [1] and coronary heart disease (CHD) [2], respectively, have been established in multiple studies over several decades and in different populations. Despite overwhelming evidence and the known epidemiological associations between short and long sleep durations and cardiometabolic disease, much less is known about the potential biological intermediates that may explain these associations. One prospective study showed that short sleep duration is associated with inflammatory markers such as C-reactive protein (CRP) [3] and interleukin 6 (IL-6) [4], and a meta-analysis found that when compared to a sleep duration of 7–8 h, sleep duration > 8 h is associated with increased CRP and IL-6 [5]. These associations are not inconsequential as systemic inflammatory markers such as CRP [6] and IL-6 [7] in turn are associated with cardiometabolic outcomes, e.g., CHD. One limitation, however, of existing studies is the high probability of reverse causality where the disease process impacts sleep. Another important limitation may be the inflammatory markers themselves; they are nonspecific to sleep duration or the health outcome and thus offer only limited information about possible pathways that link sleep duration with adverse health outcomes.

The advent of proteomic assays allows for a new approach to investigate potential biological pathways through which short or long sleep durations are associated with cardiometabolic outcomes. We have previously identified that plasma concentrations of caspase 8, an enzyme in the tumor necrosis factor receptor pathway, is associated with short sleep duration and incident DM [8]. An important recent study [9] further found that five proteomic markers related to cardiovascular risk were associated with sleep duration. Unfortunately, this study could not further investigate the prospective association between the identified proteomic markers and disease outcomes.

The aim of the present study is to identify proteomic markers associated with specific sleep duration categories. The secondary aim is to create proteomic sleep scores using the proteomic markers predictive of each sleep duration category and to further investigate the associations between the proteomic sleep scores with incident DM and incident CHD, respectively. We hypothesize that the proteomic sleep scores will be associated with both incident DM and incident CHD. Moreover, we hypothesize that the proteomic scores will abrogate any association between their corresponding phenotype and said outcomes. The proteomic scores are thus hypothesized to be on the pathway between aberrant sleep duration and incident DM and incident CHD, respectively.

## Methods

The Malmö Diet and Cancer (MDC) study is a population-based, prospective study in the city of Malmö, Sweden. Men and women aged 45–73 years were randomly selected between the years 1991 and 1996 and recruited for a baseline examination. Participants provided anthropometric data and blood samples and answered a detailed lifestyle questionnaire on heredity, socioeconomic variables, social network, occupation, physical activity, alcohol consumption, smoking, diseases, and medication. Details of the study have been described elsewhere [10]. Between 1991 and 1994, 6103 individuals from the MDC study were randomly selected to participate in the MDC Cardiovascular Cohort (MDC-CC). The purpose of the MDC-CC is to study the epidemiology of carotid artery disease [11]. MDC-CC participants underwent detailed examinations, including ultrasonography of the carotid artery, and provided plasma for the measure of novel proteomic markers.

**Fig. 1:**
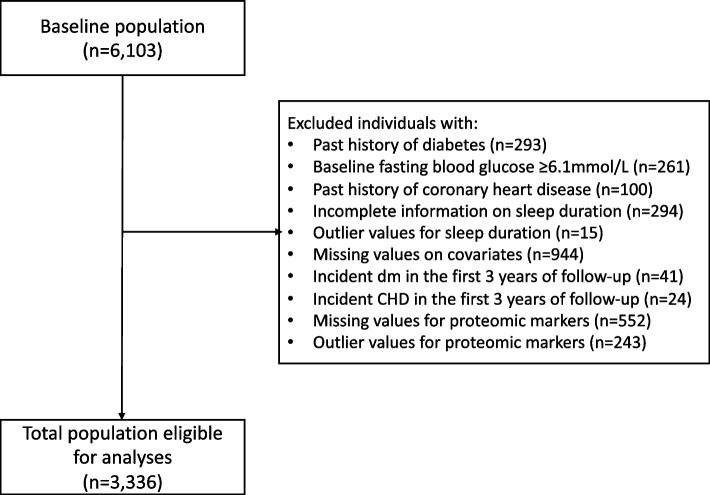
Flowchart of the inclusion and exclusion of study participants

For the purpose of the present study, MDC-CC participants were excluded if they had prevalent DM (*n* = 293) or a fasting whole blood glucose concentration ≥ 6.1 mmol/l (*n* = 261) or prevalent CHD (*n* = 100) at baseline (Fig. 1). Prevalent DM was defined as: having a measured fasting whole blood glucose ≥ 6.1 mmol/l (corresponding to fasting plasma glucose concentration ≥ 7 mmol/l) at the MDC baseline examination, a self-reported history of physician diagnosed DM, the use of DM medication according to the MDC baseline questionnaire, or being diagnosed and registered in any of the local or national diabetes registries as described elsewhere [12]. Participants were further excluded if they had provided incomplete information on sleep duration (*n* = 294) or if their sleep duration represented outlier values of more than 3 interquartile ranges below or above the first and fourth quartiles, respectively (*n* = 15), had missing data on kidney function, as measured by cystatin C (*n* = 677), or missing data for any of the covariates (*n* = 267). In order to minimize reverse causation bias, participants who were diagnosed with incident DM (*n* = 41) or incident CHD (*n* = 24) in the first 3 years of follow-up were also excluded. Finally, those with missing data for any of the proteomic markers (*n* = 552) or who had levels of proteomic markers that represented outlier values of more than 3 interquartile ranges below or above the first and fourth quartiles, respectively (*n* = 243), were also excluded. The final sample for analyses consisted of 3336 participants. All participants were followed from starting point until December 31, 2018, with person-years calculated from starting point to incident DM and incident CHD, date of death, or end of follow-up period, whichever came first. Participants who were diagnosed with both incident DM and incident CHD were included in the analyses of both outcomes unless the incident CHD event was fatal while preceding incident DM.

The MDC study was approved by the ethics committee at Lund University, and all participants provided written informed consent.

### Proteomic markers

The 92 proteomic markers investigated in this study were from the Olink Proseek Multiplex CVD 1 panel (Additional file 1: Table S1). The proteomic markers were measured in stored fasting plasma specimens from the MDC-CC baseline examination. The specimens were immediately frozen to − 80 °C following collection. Plasma concentrations of the proteomic markers were quantified using a validated high-specificity immunoassay, the Proximity Extension Assay (PEA), which has been described in detail elsewhere [13, 14].

Concentrations of proteomic markers were provided on a logarithmic (log2) scale. Any markers that were below the limit of detection were provided a missing value. For the purpose of this study, 14 protein markers with missing information for ≥ 10% (*n* = 413) of the study population were excluded from analyses (details in Additional file 1: Table S1). The concentrations of the 78 proteomic markers included in analyses were standardized, where the standard score of each marker represented the number of standard deviations above or below the mean.

### Sleep duration

Habitual sleep duration was assessed through two open questions asking participants how long they slept on weekdays and weekends, respectively. The two questions were formulated as: (a) *“How many hours do you usually sleep per night during a typical week (Monday-Friday)?”* and (b) *“How many hours do you usually sleep per night during a typical weekend (Saturday-Sunday)?”* A weighted average sleep duration [((weekday × 5) + (weekend × 2))/7] was calculated for all participants, thereby allowing the subsequent construction of a categorical variable representing sleep duration quintiles (Quintile 1 [Q1]: 4.00–6.57 h; Q2: 6.64–7.14 h; Q3: 7.21–7.57 h; Q4; 7.64–8.00 h; Q5: 8.14–11.00 h). Q3 was chosen as the reference category for two main reasons: first, the sleep duration range in this group was most similar to other studies which often use 7–8 h as the reference, thus facilitating comparisons and second, selection of the mid-quintile allowed for the investigation of J-shaped or U-shaped associations that are often reported between sleep duration and the respective outcomes e.g., [2, 15].

### Incident diabetes mellitus

Incident DM was defined as new onset DM in individuals without prevalent DM at the MDC baseline examination. All incident DM events were identified through linkage of a 10-digit national personal identification number with 6 local and national registers: the Malmö HbA_1c_ register, the Regional Diabetes 2000 register of the Scania region [16], the Swedish National Diabetes Register [17], the Swedish National Inpatient Register [18], the Swedish Cause of Death Register [19], and the Swedish Prescribed Drug Register [20]. Participants with incident DM could also be identified by having a fasting plasma glucose concentration ≥ 7 mmol/l or a 120-min plasma glucose value of > 11.0 mmol/l in subpopulations of the MDC participating in a MDC re-examination [21] or the Malmö Preventive Project re-examination [22]. The detailed process of DM end-point retrieval has been described elsewhere [23].

### Incident coronary heart disease

Incident CHD event was defined as a first fatal or non-fatal myocardial infarction, coronary artery bypass graft (CABG), or percutaneous coronary intervention (PCI). All incident CHD events were identified through linkage of a ten-digit national personal identification number with three registries validated for classification of outcomes as described elsewhere [24, 25]: the Swedish National Discharge Registry, the Swedish National Cause of Death Registry, and the Swedish Coronary Angiography and Angioplasty Registry. CABG and PCI were classified using the national classification of surgical procedures operation codes (KKÅ or Op6): 3065, 3066, 3068, 3080, 3092, 3105, 3127, 3158 for CABG, and FNG02 and FNG05 for PCI. Coronary event was defined according to the International Classification of Diseases, ninth (ICD-9) and tenth (ICD-10) revisions with fatal or non-fatal myocardial infarction (MI) or death due to CHD corresponding to codes 410, 412, and 414 (ICD-9), and I21-I23, and I25 (ICD-10).

### Covariates

Covariates in the statistical models included age at baseline (continuous), sex, cystatin C (continuous in mg/l), education (elementary school or higher than elementary school), and physical activity defined as quartiles of leisure time physical activity based on 18 items adapted from the Minnesota Leisure Time Physical Activity instrument which has been described in detail elsewhere [26]. Smoking was defined as never, past, and current smoker (< 20 cig./day, or ≥ 20 cig./day); alcohol consumption was considered as quartiles with non-consumption as the reference value (none, 0.02–1.70, 1.70–6.87, 6.88–14.36, or > 14.36 g ethanol/day), and shift work was a binary variable (yes/no). Symptoms of insomnia were based on four items: difficulty initiating sleep, difficulty maintaining sleep, early morning awakening, and not feeling rested after sleep; the score of each included item (0–3 points) were summed to an overall discrete insomnia score (0–12 points). Body mass index (BMI) was considered in categories (< 18.5 kg/m^2^, 18.5–24.9 kg/m^2^, 25.0–29.9 kg/m^2^, or ≥ 30.0 kg/m^2^). Waist circumference (in cm), low-density lipoprotein cholesterol (LDL-C, in mmol/l), high-density lipoprotein cholesterol (HDL-C, in mmol/l), triglycerides (TG, in mmol/l), and hemoglobin A1c (HbA_1c_, in %) were all treated as continuous variables. HbA_1c_ was used as a stratifying variable given that its inclusion as a covariate violated the assumptions of proportional hazards in semi-parametric survival analyses with incident DM as the outcome.

### Statistical analyses

Several of the proteomic markers in the Olink panels are expected to be highly correlated. Multicollinearity of predictors necessitates a model selection procedure retaining only markers that are relevant for the outcome of interest and have the largest effect. Lasso, least absolute shrinkage and selection operator, is an analysis method that produces sparse models by shrinking the coefficients of some predictors while setting the coefficients of other predictors to “0” [27]. This allows for improved prediction accuracy and improved interpretation [28]. However, lasso does not provide accurate standard errors of its estimate [27] which in turn precludes significance testing [29]. Cross-fit partialing-out lasso, also known as double machine learning [30], is a method that produces both coefficients and standard errors of predictors while controlling for relevant covariates. Cross-fit partialing out lasso splits samples into multiple folds selecting covariates and estimating post-selection coefficients [31]. The final results are obtained by averaging the results of multiple estimates acquired across the folds [30].

Ten-fold cross-fit partialing-out lasso logistic regression controlling for age and sex was used to estimate beta coefficients, standard errors, and *p*-values for each of the 78 proteomic markers’ associations with specific quintiles of sleep duration. Each tenfold cross-fit partialing-out lasso logistic regression was performed against a binary outcome variable where sleep duration Q3 was considered the reference value and where, in four separate analyses, the remaining four sleep duration quintiles were considered the respective outcomes of interest, i.e., cross-fit lasso 1: Q1 vs. Q3; cross-fit lasso 2: Q2 vs. Q3; cross-fit lasso 3: Q4 vs. Q3; and cross-fit lasso 4: Q5 vs. Q3. Proteomic markers that were significantly associated with Q1, Q2, Q4, and Q5, respectively, were retained (*p* for retention: *p* < 0.05). Using their beta coefficients as weights, the retained proteomic markers were then used to create four separate proteomic scores, one for each sleep quintile compared to Q3.

The four proteomic scores were included as independent variables in linear regression analyses with continuous sleep duration as the dependent variable. All possible combinations (*n* = 15) of the 4 proteomic scores were considered and each model was compared to the remaining models using the Akaike Information Criterion (AIC). The proteomic scores from the model with the lowest AIC were retained for the semi-parametric survival analyses.

Cox proportional hazards regression was used to determine hazard ratios (HR) and 95% confidence intervals (CI) for the associations of sleep duration and proteomic risk scores with incident DM and incident CHD, respectively. Primary analyses investigated the association between sleep duration with the respective outcomes. Secondary analyses considered identical models to the primary analyses with proteomic risk scores as additional main exposures, i.e., the models were adjusted for both sleep duration and proteomic score. All statistical models were stratified by HbA_1c_ using the Stata option “strata()” to account for different baseline hazards across concentrations of HbA_1c_. The minimally adjusted model (model 1) was adjusted for age, and sex; model 2 was additionally adjusted for cystatin C, education, physical activity, smoking, alcohol consumption, shift work, and symptoms of insomnia; and model 3 was additionally adjusted for BMI, waist circumference, LDL-C, HDL-C, and TG. No sex-specific stratification was done as there were no significant interactions between sex and sleep duration.

The HR of proteomic sleep duration scores are expressed in terms of incremental increases per SD.

Global tests for proportionality were conducted for the final multivariable models and did not reveal any significant deviation from the proportional hazards assumption.

A proteomic score was considered a probable mediator for the association between sleep duration and incident DM or incident CHD if the following conditions were met: (1) the inclusion of the proteomic score in the final multivariable model abrogated any significant associations between sleep duration and the outcome and (2) the proteomic score was significantly associated with the outcome. Mediating effects (survival functions for the follow-up period and the proportion mediated including 95% CI) were determined using the Stata “standsurv” post-estimation command on a fitted flexible parametric survival model that included only the main exposure (the relevant sleep duration quintile) and the probable mediator (protein score). The fitted models used three degrees of freedom for the baseline hazard and considered the sleep duration quintile as a time varying effect with three degrees of freedom.

All statistical analyses were performed using Stata/MP 17.0 (StataCorp LP, College Station, TX). The significance level was set as *p* < 0.05.

### Data sharing statement

Due to ethical and legal restrictions related to the Swedish Biobanks in Medical Care Act (2002:297) and the Personal Data Act (1998:204), data are available upon request from the data access group of Malmö Diet and Cancer study by contacting Anders Dahlin (anders.dahlin@med.lu.se).

### Role of the funding source

The sponsors had no role in study design; in the collection, analysis, and interpretation of data; in the writing of the report; or in the decision to submit the paper for publication.

## Results

Table 1 shows baseline characteristics of participants according to quintiles of habitual sleep duration. Individuals in quintile 3 (Q3; referent category in survival analyses) had the lowest average age, score of insomnia symptoms, and waist circumference. They further had the lowest proportions of participants in the highest physical activity level, non-consumers of alcohol and shift workers, and the highest proportions of participants with education greater than elementary school and with high alcohol intake. Individuals with the shortest habitual sleep duration (Q1) had the highest proportion of non-consumers of alcohol, the highest insomnia score, and the highest LDL-C.

**Table 1:** Baseline characteristics according to habitual sleep duration quintiles

Variable	Sleep duration quintile (Q; hours)					*p*-value^a^
	Q1 (4.00–6.57)	Q2 (6.64–7.14)	Q3 (7.21–7.57)	Q4 (7.64–8.00)	Q5 (8.14–11.00)	
Number of individuals	715	668	804	764	435	
Proportion of population (%)	21.4	18.5	24.1	22.9	13.0	
Age [mean (years ± s.d.)]	57.1 ± 5.8	58.5 ± 5.9	55.3 ± 5.5	58.5 ± 5.9	57.3 ± 5.9	< 0.001
Men (%)	36.5	38.7	38.6	35.2	32.2	0.15
Cystatin C [mean (mg/L ± s.d.)]	0.780 ± 0.133	0.774 ± 0.120	0.754 ± 0.124	0.773 ± 0.129	0.766 ± 0.125	0.001
Education (%)						0.003
Higher than elementary school	56.5	55.2	61.9	52.8	53.3	
Physical activity (%)						0.05
Quartile 1 (low physical activity)	26.0	23.8	24.3	24.2	27.4	
Quartile 2	22.9	26.5	29.1	23.4	25.1	
Quartile 3	24.3	24.3	26.0	24.4	22.1	
Quartile 4 (high physical activity)	26.7	25.4	20.7	28.0	25.5	
Smoking status (%)						0.44
Never	40.1	43.5	43.7	45.0	39.5	
Past	33.7	32.0	30.6	31.9	34.3	
Current < 20 cigarettes/day	13.6	12.3	14.3	11.9	11.3	
Current ≥ 20 cigarettes/day	12.6	12.1	11.4	11.1	14.9	
Alcohol consumption (%)						0.03
None	18.0	15.7	12.2	16.1	17.2	
0.02–1.70 g ethanol/day	10.8	9.7	9.8	10.7	12.2	
1.70–6.87 g ethanol/day	22.5	25.2	22.6	27.4	25.3	
6.89–14.36 g ethanol/day	24.5	23.6	27.1	24.6	22.5	
≥ 14.36 g ethanol/day	24.2	25.7	28.2	21.2	22.8	
Shift work (%)	28.8	30.1	22.8	27.1	32.2	0.002
Insomnia symptoms [median (score, iqr.)]	4 (5)	3 (4)	2 (3)	3 (4)	3 (3)	< 0.001
Waist circumference [mean (cm ± s.d.)]	82.9 ± 12.7	83.4 ± 12.0	81.6 ± 11.5	82.1 ± 12.1	81.9 ± 12.3	0.048
LDL-C [mean (mmol/L ± s.d.)]	4.3 ± 1.0	4.2 ± 0.9	4.1 ± 1.0	4.2 ± 1.0	4.1 ± 0.9	0.01
HDL-C [mean (mmol/L ± s.d.)]	1.4 ± 0.4	1.4 ± 0.4	1.4 ± 0.4	1.4 ± 0.4	1.4 ± 0.4	0.89
Triglycerides [mean (mmol/L ± s.d.)]	1.3 ± 0.6	1.3 ± 0.6	1.3 ± 0.6	1.3 ± 0.6	1.3 ± 0.7	0.81
HbA1c [mean (% ± s.d.)]	4.8 ± 0.4	4.8 ± 0.4	4.7 ± 0.4	4.8 ± 0.4	4.8 ± 0.4	0.14
BMI						0.16
< 18.5 kg/m^2^	1.1	1.0	0.6	1.1	0.7	
18.5–25.0 kg/m^2^	47.7	45.3	52.4	48.4	49.4	
25.0–30.0 kg/m^2^	38.6	42.2	39.3	39.1	38.6	
≥ 30.0 kg/m^2^	12.6	11.5	7.7	11.4	11.3	

*BMI* body mass index, *iqr* interquartile range, *s.d.* standard deviation

^a^Chi-square test for categorical variables, ANOVA for continuous variables (age, cystatin C, waist circumference, low-density lipoprotein cholesterol, high-density lipoprotein cholesterol, triglycerides, and hemoglobin A1c ), Kruskal–Wallis test for insomnia symptoms

Additional file 1: Table S2 shows the beta coefficients, standard errors, z-scores, p-values, and 95% confidence intervals of all 78 proteomic markers for their association with each sleep duration quintile when compared to sleep duration Q3 using the cross-fit partialing-out lasso logistic regression. A total of 16 unique proteomic markers were significantly associated with sleep duration quintiles Q1, Q2, Q4, and Q5 when compared to Q3 (Table 2); 6 proteomic markers were significantly associated with sleep duration Q1; 4 proteomic markers were significantly associated with Q2; 5 proteomic markers were significantly associated with Q4; and 6 proteomic markers were significantly associated with Q5. Thirteen of the markers were significantly associated only with one specific sleep quintile; 1 proteomic marker was associated with both short sleep duration quintiles (Q1 and Q2); and 2 proteomic markers were associated with both short and long sleep duration quintiles.

**Table 2:** Proteomic markers significantly associated with specific sleep duration quintiles when compared to referent sleep duration (quintile 3) using tenfold cross-fit partialing out lasso logistic regression analyses

Sleep duration quintile	Proteomic marker	Beta coefficient	SE	*z*	*p*-value	95% confidence interval
Quintile 1 vs Quintile 3	TRANCE	− 0.199	0.084	− 2.37	0.018	− 0.363 to − 0.035
	MMP-7	0.193	0.082	2.37	0.018	0.033 to 0.353
	MMP-10	− 0.160	0.069	− 2.32	0.020	− 0.295 to − 0.025
	Follistatin	0.206	0.091	2.25	0.024	0.027 to 0.385
	E-selectin	0.201	0.091	2.22	0.027	0.023 to 0.378
	TRAIL-R2	0.266	0.133	2.00	0.046	0.005 to 0.528
Quintile 2 vs Quintile 3	TRAIL-R2	0.431	0.143	3.02	0.0026	0.151 to 0.711
	FAS	− 0.457	0.156	− 2.92	0.0035	− 0.763 to − 0.150
	Kallikrein-6	− 0.284	0.111	− 2.57	0.010	− 0.500 to − 0.067
	U-PAR	− 0.302	0.140	− 2.16	0.031	− 0.576 to − 0.0280
Quintile 4 vs Quintile 3	FAS	− 0.452	0.135	− 3.34	0.00084	− 0.717 to − 0.187
	Renin	0.186	0.070	2.64	0.0082	0.048 to 0.324
	HB-EGF	− 0.329	0.135	− 2.44	0.015	− 0.594 to − 0.064
	MMP-7	0.189	0.081	2.34	0.019	0.031 to 0.348
	CXCL6	0.210	0.097	2.16	0.031	0.019 to 0.400
Quintile 5 vs Quintile 3	Prolactin	0.261	0.081	3.22	0.0013	0.102 to 0.420
	MMP-7	0.296	0.097	3.05	0.0023	0.106 to 0.486
	CXCL1	0.245	0.100	2.44	0.015	0.0483 to 0.441
	FAS	− 0.402	0.170	− 2.37	0.018	− 0.735 to − 0.070
	tPA	0.256	0.110	2.32	0.020	0.0395 to 0.472
	HSP27	− 0.284	0.130	− 2.19	0.029	− 0.538 to − 0.030

*TRANCE* tumor necrosis factor-related activation-induced cytokine, *MMP-7* matrix metalloproteinase-7, *MMP-10* matrix metalloproteinase-10, *TRAIL-R2* tumor necrosis factor-related apoptosis-inducing ligand receptor 2, *FAS* tumor necrosis factor receptor superfamily member 6, *U-PAR* urokinase plasminogen activator surface receptor, *HB-EGF* proheparin-binding epidermal growth factor-like growth facto, *CXCL6* C-X-C motif chemokine 6, *CXCL1* C-X-C motif chemokine 1, *tPA* tissue-type plasminogen activator, *HSP27* heat shock 27 kDa protein

Linear regression analyses of the 15 combinations of the 4 proteomic scores (4 models including 1 score each; 6 models combining variations of 2 scores; 4 models combining variations of 3 scores; and 1 model including all scores) revealed that the model that included proteomic scores for Q1 and Q5 had the lowest AIC and thus best predicted sleep duration (data not shown).

### Sleep duration, proteomic scores, and incident diabetes mellitus

Mean follow-up time for incident DM was 21.8 years (72,565 person-years) during which there were 522 cases of incident DM. In the age- and sex adjusted model (model 1), all sleep duration quintiles were significantly and positively associated with incident DM when compared with Q3 (Q1: hazard ratio [HR] = 1.41, 95% CI: 1.08–1.84; Q2: HR = 1.40, 95% CI: 1.06–1.85; Q4: HR = 1.32, 95% CI: 1.01–1.72; Q5: HR = 1.50, 95% CI: 1.11–2.03) (Table 3). The associations were only minimally attenuated in model 2, with all but one sleep duration quintile remaining significantly associated with incident DM in model 3 (Q1: HR = 1.32, 95% CI: 1.00–1.74; Q2: HR = 1.33, 95% CI: 1.00–1.76; Q5: HR = 1.48, 95% CI: 1.09–2.00). The addition of proteomic scores for sleep duration Q1 and Q5 attenuated the associations in model 1; in models mutually adjusted for sleep duration and proteomic scores, sleep duration Q1 (HR = 1.33, 95% CI: 1.02–1.74), Q2 (HR = 1.34, 95% CI: 1.01–1.77), and Q5 (HR = 1.46, 95% CI: 1.08–1.97) were significantly associated with incident DM (Table 4). The addition of covariates in model 2 abrogated the association between sleep duration Q1 and incident DM and attenuated the association between sleep duration Q5 and incident DM. In model 3, only sleep duration Q5 remained significantly and positively associated with incident DM (HR = 1.42, 95% CI: 1.04–1.93). The proteomic score for sleep duration Q1 was attenuated with the inclusion of covariates in models 2 and 3 but remained significantly and positively associated with incident DM in all models (model 1, proteomic score Q1: HR = 1.52, 95% CI: 1.28–1.81; model 2, proteomic score Q1: HR = 1.48, 95% CI: 1.24–1.77; model 3, proteomic score Q1: HR = 1.27, 95% CI: 1.06–1.53). The proteomic score for sleep duration Q5 was significantly and positively associated with incident DM in model 1 (HR = 1.22, 95% CI: 1.01–1.48) and model 2 (HR = 1.25, 95% CI: 1.03–1.52), but was abrogated following the inclusion of covariates in model 3 (HR = 1.20, 95% CI: 0.99–1.45).

**Table 3:** Cox proportional hazards regression models for the association between sleep duration and incident diabetes mellitus

	Model 1		Model 2		Model 3	
	HR	95% CI	HR	95% CI	HR	95% CI
Sleep duration Q1 (4.00–6.57 h)	1.41*	(1.08, 1.84)	1.32*	(1.01, 1.74)	1.32*	(1.00, 1.74)
Sleep duration Q2 (6.64–7.14 h)	1.40*	(1.06, 1.85)	1.40*	(1.05, 1.85)	1.33*	(1.00, 1.76)
Sleep duration Q3 (7.21–7.57 h)	Reference		Reference		Reference	
Sleep duration Q4 (7.64–8.00 h)	1.32*	(1.01, 1.72)	1.31*	(1.00, 1.72)	1.30	(0.99, 1.70)
Sleep duration Q5 (8.14–11.00 h)	1.50**	(1.11, 2.03)	1.47*	(1.09, 1.99)	1.48*	(1.09, 2.00)

Italic values denote statistically significant results

Model 1 is stratified by HbA1c concentration and adjusted for age and sex

Model 2 is additionally adjusted for cystatin C, education, physical activity, smoking, alcohol consumption, shift work, and insomnia symptoms

Model 3 is additionally adjusted for body mass index, waist circumference, low-density lipoprotein cholesterol, high-density lipoprotein cholesterol, and triglycerides

*p < 0.05, **p < 0.01

**Table 4:** Cox proportional hazards regression models for the association between proteomic sleep score and sleep duration with incident diabetes mellitus. Analyses are mutually adjusted for proteomic sleep score and sleep duration

	Model 1		Model 2		Model 3	
	HR	(95% CI)	HR	95% CI	HR	95% CI
Proteomic score Q1^a^	1.52***	*(1.28, 1.81)*	1.48***	*(1.24, 1.77)*	1.27**	*(1.06, 1.53)*
Proteomic score Q5^a^	1.22*	*(1.01, 1.48)*	1.25*	*(1.03, 1.52)*	1.20	(0.99, 1.45)
Sleep duration Q1 (4.00–6.57 h)	1.33*	(1.02, 1.74)	1.26	(0.96, 1.66)	1.27	(0.96, 1.67)
Sleep duration Q2 (6.64–7.14 h)	1.34*	(1.01, 1.77)	1.34*	(1.01, 1.77)	1.29	(0.97, 1.71)
Sleep duration Q3 (7.21–7.57 h)	Reference		Reference		Reference	
Sleep duration Q4 (7.64–8.00 h)	1.27	(0.97, 1.66)	1.26	(0.96, 1.65)	1.25	(0.95, 1.64)
Sleep duration Q5 (8.14–11.00 h)	1.46*	(1.08, 1.97)	1.42*	(1.05, 1.93)	1.42*	(1.04, 1.93)

Italic values denote statistically significant results

Model 1 is stratified by HbA1c concentration and adjusted for age and sex

Model 2 is additionally adjusted for cystatin C, education, physical activity, smoking, alcohol consumption, shift work, and insomnia symptoms

Model 3 is additionally adjusted for body mass index, waist circumference, low-density lipoprotein cholesterol, high-density lipoprotein cholesterol, and triglycerides

^a^Expressed as the HR and 95% CI of the incremental increase per SD of the proteomic sleep score

^*^p < 0.05, **p < 0.01, ***p < 0.001

**Fig. 2:**
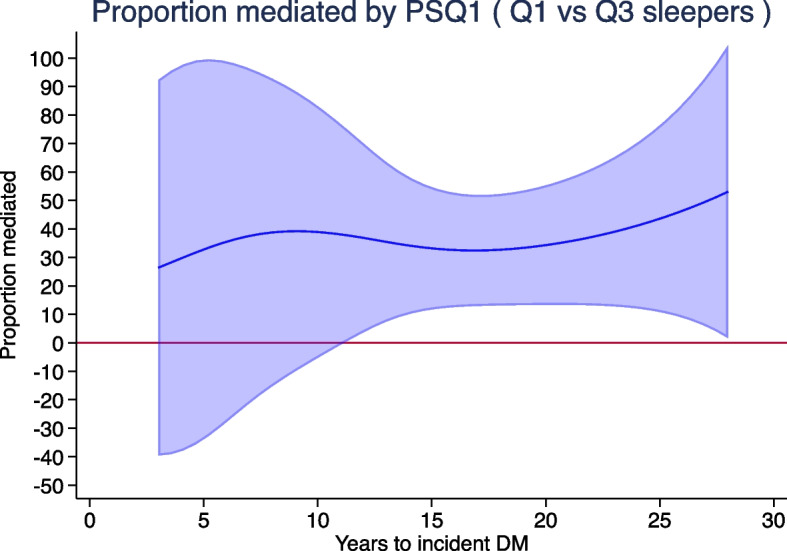
Proportion of the association between sleep duration Q1 and incident diabetes mellitus (DM) mediated by the proteomic risk score for sleep duration Q1 (PSQ1). Light blue area indicates 95% confidence intervals; horizontal line is a reference indicator for 0% mediation. Confidence intervals above the reference line represent statistically significant mediation

Mediation analyses found that when compared to referent sleep duration Q3, the proteomic risk score for sleep duration Q1 significantly mediated between 32 and 53% of the association between sleep duration Q1 and incident DM during follow-up years 11.1 to 27.2 (Fig. 2). The protein score was not a significant mediator during follow-up years 3.0 to 11.0.

### Sleep duration, proteomic scores, and incident coronary heart disease

Mean follow-up time for incident CHD was 22.4 years (74,681 person-years) during which there were 411 cases of incident CHD. In the age- and sex adjusted model (model 1), sleep duration Q1 and Q2 were significantly and positively associated with incident CHD when compared to Q3 (Q1: HR = 1.53, 95% CI: 1.14–2.06; Q2: HR = 1.37, 95% CI: 1.01–1.88) (Table 5). The association between sleep duration Q2 and incident CHD was abrogated in model 2 and model 3, whereas the association between sleep duration Q1 and incident CHD was only minimally attenuated in model 2 (HR = 1.39, 95% CI: 1.02–1.88) and model 3 (HR = 1.37, 95% CI: 1.01–1.86). The addition of proteomic scores of sleep durations Q1 and Q5 (i.e., models mutually adjusted for sleep duration and proteomic scores) abrogated the association between sleep duration Q2 and incident CHD in model 1, whereas sleep duration Q1 remained significantly associated with incident CHD in all models (model 1: HR = 1.51, 95% CI: 1.12–2.04; model 2: HR = 1.38, 95% CI: 1.02–1.88; model 3: HR = 1.37, 95% CI: 1.01–1.86) (Table 6). Proteomic scores for sleep durations Q1 and Q5 were not significantly associated with incident CHD in any model.

**Table 5:** Cox proportional hazards regression models for the association between sleep duration and incident coronary heart disease

	Model 1		Model 2		Model 3	
	HR	95% CI	HR	95% CI	HR	95% CI
Sleep duration Q1 (4.00–6.57 h)	1.53**	(1.14, 2.06)	1.39*	(1.02, 1.88)	1.37*	(1.01, 1.86)
Sleep duration Q2 (6.64–7.14 h)	1.37*	(1.01, 1.88)	1.34	(0.98, 1.84)	1.35	(0.99, 1.85)
Sleep duration Q3 (7.21–7.57 h)	Reference		Reference		Reference	
Sleep duration Q4 (7.64–8.00 h)	1.21	(0.89, 1.64)	1.20	(0.88, 1.64)	1.22	(0.89, 1.66)
Sleep duration Q5 (8.14–11.00 h)	1.24	(0.86, 1.78)	1.19	(0.83, 1.72)	1.23	(0.85, 1.77)

Italic values denote statistically significant results

Model 1 is stratified by HbA1c concentration and adjusted for age and sex

Model 2 is additionally adjusted for cystatin C, education, physical activity, smoking, alcohol consumption, shift work, and insomnia symptoms

Model 3 is additionally adjusted for body mass index, waist circumference, low-density lipoprotein cholesterol, high-density lipoprotein cholesterol, and triglycerides

^*^p < 0.05, **p < 0.01

**Table 6:** Cox proportional hazards regression models for the association between proteomic sleep score and sleep duration with incident coronary heart disease. Analyses are mutually adjusted for proteomic sleep score and sleep duration

	Model 1		Model 2		Model 3	
	Coefficient	95% CI	Coefficient	95% CI	Coefficient	95% CI
Proteomic score Q1^a^	1.13	(0.93, 1.38)	1.03	(0.84, 1.25)	1.03	(0.84, 1.26)
Proteomic score Q5^a^	1.00	(0.80, 1.24)	1.02	(0.82, 1.26)	1.02	(0.82, 1.26)
Sleep duration Q1 (4.00–6.57 h)	1.51**	(1.12, 2.04)	1.38*	(1.02, 1.88)	1.37*	(1.01, 1.86)
Sleep duration Q2 (6.64–7.14 h)	1.36	(0.99, 1.86)	1.34	(0.98, 1.83)	1.35	(0.98, 1.85)
Sleep duration Q3 (7.21–7.57 h)	Reference		Reference		Reference	
Sleep duration Q4 (7.64–8.00 h)	1.20	(0.88, 1.63)	1.20	(0.88, 1.63)	1.21	(0.89, 1.66)
Sleep duration Q5 (8.14–11.00 h)	1.24	(0.86, 1.78)	1.19	(0.82, 1.71)	1.22	(0.85, 1.77)

Italic values denote statistically significant results

Model 1 is stratified by HbA1c concentration and adjusted for age and sex

Model 2 is additionally adjusted for cystatin C, education, physical activity, smoking, alcohol consumption, shift work, and insomnia symptoms

Model 3 is additionally adjusted for body mass index, waist circumference, low-density lipoprotein cholesterol, high-density lipoprotein cholesterol, and triglycerides

^a^Expressed as the HR and 95% CI of the incremental increase per SD of the proteomic sleep score

^*^p < 0.05,**p < 0.01

## Discussion

The key results of this study are first, sleep duration as the single main exposure is associated with both incident DM and incident CHD. Second, 16 unique proteomic markers significantly predict specific sleep duration quintiles when compared to referent sleep duration. Third, proteomic scores, created using the weights of individual proteomic markers, were differentially associated with incident DM and incident CHD thereby suggesting independent pathways for the association between sleep duration with specific cardiometabolic diseases. Fourth, the proteomic score for sleep duration Q1 was a significant mediator of the association between sleep duration Q1 and incident DM. The results are discussed in detail below.

### Association between sleep duration and cardiometabolic disease

The association between sleep duration and incident DM indicate an approximately 30% increased risk of DM with sleep durations shorter than the referent 7.21–7.57 h and an increased risks of approximately 50% for those with sleep durations 8.14–11.00 h. These results and the J-shaped association are in line with our hypothesis and consistent with several studies published to date, including a large meta-analysis [1]. One major difference, however, when comparing with other studies are the similar risks seen in this study for Q1 (HR: 1.32) and Q2 (HR: 1.33) when compared with Q3. The most probable explanation for this is the decision to, in the present study, use statistically defined cutoffs (i.e., quintiles) rather than discrete hourly cutoffs, in order to get similarly sized groups. This approach may dilute the relative risks in the shortest sleep duration quintile given that the reference category is approximately 26 min short of 8 h which in many studies is considered referent sleep duration. Despite this, the findings confirm the independent and positive association between both short (Q1 and Q2) and long (Q5) sleep durations with incident DM.

The association between sleep duration and incident CHD indicates a 37% increased risk of CHD only for those with the shortest sleep duration (Q1) when compared to Q3. These results are very similar to our findings from a larger sample of the MDC cohort which showed 41% increased risk of CHD for men (*n* = 6966) and 46% increased risk for women (*n* = 9378) when comparing < 6 h of sleep with a referent 7–8 h [32]. Our obtained results for the association between short sleep duration and CHD are also confirmed by meta-analyses [2, 33]. The absence of any association between the longest sleep quintile (Q5) with CHD in the present study could be explained by the decision to not stratify by sex. Indeed, in a larger sample from the same cohort, sleep duration ≥ 9 h was associated with a 33% increased risk of incident CHD among men but not women. Another possible explanation for this discrepancy could be the categorization of sleep duration into quintiles where Q5 represents a relatively broad sleep duration range (8.14–11.0 h). The National Sleep Foundation guidelines recommend a sleep duration of 7–9 h [34].

### Proteomic markers predictive of sleep duration

Sixteen unique proteomic markers were found to be significantly associated with sleep duration when comparing four sleep duration quintiles to referent sleep duration (Q3). The combination of proteomic scores for Q1 and Q5 best predicted sleep duration.

The proteomic score for sleep duration Q1 consisted of six unique proteomic markers, however, the scientific literature reveals very little about how these proteomic markers and their corresponding proteins are related to sleep duration. Of the six markers, only two have previous studies indicating a possible association with sleep-related outcomes; follistatin (FS), upregulated in the shortest sleep quintile in our study, has an L-shaped association with sleep duration [9], and concentrations of circulating FS suggest a circadian variation [35]. The function of circulating FS is not known but is suggested to be involved in regulating energy metabolism [36]. E-selectin, a cellular adhesion molecule [37], was upregulated in the shortest sleep quintile which is supported by at least one study in which one night of sleep deprivation was associated with increased concentrations of soluble E-selectin [38]. Moreover, E-selectin concentrations are increased in obstructive sleep apnea [39] and are reduced following treatment with continuous positive airway pressure [39, 40]. Those findings suggest that concentrations of E-selectin may rapidly adjust with changes to sleep dynamics. It should be noted that two studies found no significant association between sleep duration and E-selectin [41, 42]. For the remaining four markers that were associated with the shortest sleep duration quintile in this study, the literature, to the best of our knowledge, reveals no findings linked to sleep duration or sleep-related outcomes. In the present study, tumor necrosis factor-related activation-induced cytokine (TRANCE), also known as receptor activator of nuclear factor-kb ligand (RANKL), as well as matrix metalloproteinase (MMP)-10 were downregulated, whereas MMP-7 and tumor necrosis factor-related apoptosis-inducing ligand receptor 2 (TRAIL-R2) were upregulated with the shortest sleep duration quintile. RANKL has been implicated in vascular inflammation, vascular calcification, and angiogenesis [43]; MMP-10 is involved in the resolution of acute inflammation [44]; MMP-7 has been linked with both proapoptotic and cell proliferative pathways through cleavage of the ligand FasL and its involvement in the Fas/FasL pathway [45]; TRAIL-R2 triggers caspase-dependent apoptosis [46] and soluble TRAIL-R2 is released with apoptosis activated through the Fas/FasL pathway [47]. In summary, very short sleep duration is linked with upregulation of pro-inflammatory and pro-apoptotic markers and of markers related to cell adhesion and with downregulation of markers related to angiogenesis and resolution of inflammation.

The longest sleep duration quintile was positively associated with prolactin, MMP-7, C-X-C motif chemokine 1 (CXCL1), tissue-type plasminogen activator (t-PA), and inversely associated with tumor necrosis factor receptor superfamily member 6 (Fas), and heat shock 27 kDa protein (HSP 27). There is only scarce information in the existing literature linking these specific markers with sleep-related outcomes. Prolactin is involved in lactation, reproduction, immune response, and angiogenesis [48], and prolactin levels have been shown to increase during sleep [49–51], which is in line with our findings. tPA is involved in endogenous fibrinolysis [52]. Acute release of tPA in men is reduced in habitually short sleepers (< 7.0 h) compared to those who sleep between 7.0 h and 8.1 h [53]. Moreover, tPa has been found to be positively associated with moderate to severe obstructive sleep apnea (OSA) [39, 54] and with desaturation events during sleep [54]. These findings are also in line with our finding of tPA upregulation with the longest sleep quintile and may suggest a link between long self-reported sleep duration and OSA. Fas is a cell-surface receptor involved in caspase-dependent apoptosis [45, 55]. Although no studies link plasma concentrations of Fas with sleep outcomes, it is noteworthy that plasma concentrations of caspase-8 are themselves positively associated with short sleep duration [8] which would be in line with the downregulation of Fas with long sleep as found in the present study. CXCL1 is a chemoattractant cytokine that increases neutrophile recruitment in inflammation and promotes angiogenesis [56]. HSP27 functions as an antioxidant and an anti-apoptotic agent during oxidative and chemical stress, respectively [57]. Taken together, the longest sleep duration is linked with upregulation of markers related to angiogenesis, cell proliferation and fibrinolysis, and downregulation of markers related to apoptosis. The upregulation of MMP-7 with both short and long sleep duration could be due to its suggested involvement in both cell proliferative and proapoptotic pathways [45]. Based on the suggested function of the remaining proteins, it could be speculated that the role of MMP-7 in long sleep is related to cell proliferation.

### Association between proteomic scores and cardiometabolic disease

The two proteomic scores for sleep durations Q1 and Q5 together best predicted habitual sleep duration in a multilinear regression model. This result is in line with expectations given that the two scores account for the extreme sleep duration quintiles. Consequently, the two quintiles account for the largest proportion of variance explained while keeping the number of predictors in the model to a minimum. The inclusion of the two proteomic scores in the models for sleep duration and incident DM abrogated the association for sleep durations Q1 and Q2 in the final multivariable model. The proteomic score for Q1 remained significantly associated with incident DM in all models albeit with some attenuation following the inclusion of additional covariates in models 2 and 3. This would be expected given that several covariates are known to be associated with DM; there is therefore little reason to believe that that they would not be associated also with proteomic markers of inflammation. Indeed, of the six markers included in the proteomic score for sleep duration Q1, FS [58], E-selectin [59], MMP-7 [60], and TRAIL-R2 [47] have been shown to be associated with the risk of DM, while RANKL is inversely associated with prevalent DM [61].

Mediation analyses revealed that the proteomic score for sleep duration Q1 was a significant mediator of approximately 40% of the association between sleep duration Q1, when compared to Q3, and incident DM. It is noteworthy that mediation was significant after 11 years of follow-up; however, this could be explained by the comparatively small number of cases of incident DM (*n* = 121) during follow-up years 3.0–11.0 compared to the 401 cases during follow-up years 11.1–27.2. These findings strongly suggest that the proteomic score for sleep duration Q1 provides an explanation of the mechanism underlying the association between short sleep duration and incident DM and as such could be considered a proteomic fingerprint of very short sleep duration. Conversely, the proteomic score for sleep duration Q5 was only marginally attenuated by the inclusion of covariates but was itself not significantly associated with incident DM in the final model. Moreover, the corresponding sleep duration Q5 phenotype remained, with minimal attenuation, significantly associated with incident DM throughout all models. The findings thus highlight that although specific proteomic markers are predictive of the longest sleep duration, their combination does not explain the mechanism through which the longest sleep is associated with incident DM. Instead, the stable association of the longest sleep phenotype with incident DM therefore points towards residual confounding.

For the association between sleep duration and incident CHD, the inclusion of proteomic scores for sleep durations Q1 and Q5 had no effect on the association between sleep duration and incident CHD. Moreover, the proteomic markers did not attenuate the strength of the association between sleep duration Q1 and incident CHD. More surprisingly, neither of the proteomic scores were associated with incident CHD in any of the models. This is an unexpected finding that contradicts our initial hypothesis. All proteomic markers were derived from a CVD panel with the expectation that their combination would be predominantly associated with a CVD outcome. Contrary to being a limitation of the study, this result could instead be used to argue that the specific combination of markers and their respective weights is explanatory only of incident DM. Consequently, the observed association between sleep duration Q1 and incident CHD suggests that incident DM itself may be the intermediate explanatory stage. Indeed, when considering incident DM as a time-varying covariate in the present study, the association between sleep duration Q1 and incident CHD is abrogated (results not shown). This suggests an overall pathway where very short sleep duration, through inflammatory and apoptotic processes, is associated with incident DM which in turn is an intermediate step explaining the downstream association with incident CHD. The latter part confirms the results of a recent study using the same study cohort [32].

## Limitations

This study has some limitations. First, we have used only one Olink panel whereas another study [9] used multiple panels. Moreover, today’s proteomic assays allow for the quantification of several thousand proteomic markers; it is important that studies with available large-scale proteomic data aim to replicate the findings. Nevertheless, the CVD I panel used in the present study is highly relevant to cardiometabolic disease and inflammation. Second, the found association between long sleep duration and incident DM is most likely driven by other factors than inflammation. Alternatively, the association could be explained by inflammatory and cardiometabolic markers other than the ones investigated in the study. Third, there is possible circadian variation of at least two proteomic markers (FS and E-selectin) [38] indicating that timing of the blood sampling may be a relevant factor. However, all samples were taken during morning (7–11 AM) hours and despite differences in timing during that period, we would expect that this is a non-differential misclassification which drives results towards the null. Fourth, the current study does not allow for any conclusion regarding the specific tissues that may be involved in the associations between sleep duration and circulating proteomic markers. Fifth, we were not able to include OSA as a covariate. OSA is associated with several of the found markers [39, 40, 54] and could potentially be associated with additional markers. However, the role of OSA would appear to be more relevant for long sleep duration. Sixth, this study did not consider hypnotics or psychotropic drugs that may well impact the exposures and outcomes of this study. However, we adjusted for symptoms of insomnia which is prevalent in many with affective disorder and may serve as a proxy for the use of hypnotics. Seventh, due to sample size, sleep duration was categorized into quintiles to allow for relevant statistical analyses. However, larger studies with information on both sleep duration and proteomic markers would do well to also consider discrete hourly sleep duration categories. Finally, several markers (e.g., CXCL1 and MMP-7) are involved in multiple biological pathways; plasma concentrations do not elucidate the specific pathways that are involved. However, the combination of markers allows for educated speculation as to which pathways that may be relevant and is in itself a strength of the present study’s approach.

## Strengths

This study also has several strengths. First, lasso as a selection procedure allows for the inclusion of all relevant proteomic markers while avoiding multicollinearity. Indeed, it is expected that many of the included proteins represent similar or identical biological pathways and would therefore be concomitantly up- and downregulated with any specific exposure of interest. Moreover, lasso iterates over several subsets of data before estimating coefficients based on average values of the selected markers. Second, the combination of individual proteomic markers into proteomic scores has the benefit of offering a more comprehensive view of any possible underlying mechanisms of the association between sleep duration and cardiometabolic outcomes. Third, the analyses excluded the first 3 years of follow-up thereby minimizing the risk of reverse causation; indeed, any illness or disease process related to inflammation would be expected to have a direct impact on sleep duration. Fourth, participants in the study were followed for an average of approximately 22 years. Fifth, both incident DM and incident CHD were collected from highly accurate nationwide registers.

## Conclusions

This study found differential proteomic marker expressions for the very shortest and longest sleep duration quintiles in a general population cohort. The proteomic score of the shortest sleep duration was positively associated with incident DM but not with incident CHD. It is therefore suggested that the proinflammatory and proapoptotic pathways identify a biological mechanism that mediates and links very short sleep duration specifically to incident DM. No associations were found for the proteomic score of the longest sleep duration with incident DM or incident CHD thereby suggesting residual confounding as a possible explanation.

### Supplementary Information

**Additional file 1: Table S1** Proteomic markers included in the Olink Proseek Multiplex CVD 1 panel. **Table S2** Proteomic markers and their association with specific sleep duration quintiles.

## Data Availability

Due to ethical and legal restrictions related to the Swedish Biobanks in Medical Care Act (2002:297) and the Personal Data Act (1998:204), data are available upon request from the data access group of Malmö Diet and Cancer study by contacting Anders Dahlin (anders.dahlin@med.lu.se).

## Abbreviations

DM: Diabetes mellitus
CHD: Coronary heart disease
CRP: C-reactive protein
IL-6: Interleukin 6
MDC: Malmö Diet and Cancer
MDC-CC: Malmö Diet and Cancer Cardiovascular Cohort
CVD: Cardiovascular disease
PEA: Proximity extension assay
CABG: Coronary artery bypass graft
PCI: Percutaneous coronary intervention
MI: Myocardial infarction
LDL-C: Low-density lipoprotein cholesterol
HDL-C: High-density lipoprotein cholesterol
TG: Triglycerides
HbA1c: Hemoglobin A1c
AIC: Akaike Information Criterion
HR: Hazard ratios
CI: Confidence intervals
FS: Follistatin
TRANCE: Tumor necrosis factor-related activation-induced cytokine
RANKL: Receptor activator of nuclear factor-kb ligand
MMP-10: Matrix metalloproteinase-10
MMP-7: Matrix metalloproteinase-7
TRAIL-R2: Tumor necrosis factor-related apoptosis-inducing ligand receptor 2
CXCL1: C-X-C motif chemokine 1
t-PA: Tissue-type plasminogen activator
HSP 27: Heat shock 27 kDA protein
OSA: Obstructive sleep apnea
