# Supplementary material for: Very short sleep duration reveals a proteomic fingerprint that is selectively associated with incident diabetes mellitus but not with incident coronary heart disease: a cohort study
Source: BMC Med. 2024 Apr 23;22:173. doi: 10.1186/s12916-024-03392-1 (PMC11035142; doi:10.1186/s12916-024-03392-1)
Supplement: Supplementary file 1 — Additional file 1: Table S1 Proteomic markers included in the Olink Proseek Multiplex CVD 1 panel. Table S2 Proteomic markers and their association with specific sleep duration quintiles. [file 12916_2024_3392_MOESM1_ESM.docx]

Table S1. Proteomic markers included in the Olink Proseek Multiplex CVD 1 panel, their proportion of missing information and status in the final analyses

| Proteomic marker | Missing information (%) |  | Final analysis |
| --- | --- | --- | --- |
| IL-8 | 7.2% |  | Included |
| VEGF-A | 7.1% |  | Included |
| AM | 7.1% |  | Included |
| CD40-L | 7.1% |  | Included |
| GDF-15 | 7.1% |  | Included |
| PlGF | 7.1% |  | Included |
| SELE | 7.1% |  | Included |
| EGF | 7.1% |  | Included |
| OPG | 7.1% |  | Included |
| SRC | 7.1% |  | Included |
| IL-1ra | 20.7% |  | Excluded |
| IL-6 | 7.1% |  | Included |
| CSTB | 7.1% |  | Included |
| MCP-1 | 7.1% |  | Included |
| KLK6 | 7.1% |  | Included |
| Gal-3 | 7.1% |  | Included |
| PAR-1 | 7.1% |  | Included |
| TRAIL | 7.1% |  | Included |
| hK11 | 7.1% |  | Included |
| TIE2 | 7.1% |  | Included |
| TF | 7.1% |  | Included |
| TNF-R1 | 7.1% |  | Included |
| PDGF subunit B | 7.1% |  | Included |
| IL27-A | 7.1% |  | Included |
| CSF-1 | 7.1% |  | Included |
| CXCL1 | 7.1% |  | Included |
| LOX-1 | 7.1% |  | Included |
| TRAIL-R2 | 7.1% |  | Included |
| FGF-23 | 7.1% |  | Included |
| SCF | 7.1% |  | Included |
| IL-18 | 7.1% |  | Included |
| IL-6RA | 7.1% |  | Included |
| TNF-R2 | 7.1% |  | Included |
| MMP-3 | 11.4% |  | Excluded |
| HSP 27 | 7.1% |  | Included |
| TNFSF14 | 7.2% |  | Included |
| PRL | 7.2% |  | Included |
| MPO | 7.1% |  | Included |
| GH | 7.1% |  | Included |
| MMP-1 | 7.2% |  | Included |
| RETN | 7.1% |  | Included |
| FAS | 7.1% |  | Included |
| PAPPA | 11.5% |  | Excluded |
| PTX3 | 12.3% |  | Excluded |
| REN | 7.1% |  | Included |
| CHI3L1 | 7.4% |  | Included |
| ST2 | 7.2% |  | Included |
| TIM | 7.2% |  | Included |
| Beta-NGF | 89.2% |  | Excluded |
| mAmP | 28.3% |  | Excluded |
| TRANCE | 8.0% |  | Included |
| HGF | 7.1% |  | Included |
| PSGL-1 | 53.1% |  | Excluded |
| MB | 7.1% |  | Included |
| TM | 7.1% |  | Included |
| IL-16 | 7.1% |  | Included |
| MMP-10 | 7.1% |  | Included |
| Table S1 continued | |  |  |
| U-PAR | 7.1% |  | Included |
| CCL4 | 7.1% |  | Included |
| CTSD | 7.1% |  | Included |
| RAGE | 7.1% |  | Included |
| CCL3 | 8.9% |  | Included |
| MMP-7 | 7.2% |  | Included |
| CXCL6 | 7.1% |  | Included |
| ITGB1BP2 | 10.3% |  | Excluded |
| CXCL16 | 7.7% |  | Included |
| Dkk-1 | 7.1% |  | Included |
| SIRT2 | 7.2% |  | Included |
| GAL | 7.1% |  | Included |
| AGRP | 7.1% |  | Included |
| EN-RAGE | 97.7% |  | Excluded |
| CD40 | 7.1% |  | Included |
| t-PA | 7.1% |  | Included |
| HB-EGF | 7.1% |  | Included |
| ESM-1 | 7.1% |  | Included |
| IL-4 | 99.4% |  | Excluded |
| VEGF-D | 7.1% |  | Included |
| MMP-12 | 7.1% |  | Included |
| SPON1 | 7.1% |  | Included |
| CASP-8 | 8.5% |  | Included |
| CTSL1 | 7.1% |  | Included |
| CX3CL1 | 7.1% |  | Included |
| FABP4 | 10.6% |  | Excluded |
| BNP | 86.9% |  | Excluded |
| LEP | 9.2% |  | Included |
| CCL20 | 7.1% |  | Included |
| CA-125 | 14.8% |  | Excluded |
| NEMO | 7.1% |  | Included |
| FS | 7.1% |  | Included |
| PECAM-1 | 7.1% |  | Included |
| NT-pro-BNP | 22.3% |  | Excluded |
| ECP | 7.2% |  | Included |

Abbreviations: IL-8: Interleukin-8, VEGF-A: Vascular endothelial growth factor A, AM: Adrenomedullin, CD40-L: CD40 ligand, GDF-15: Growth/differentiation factor 15, PlGF: Placenta growth factor, SELE: E-selectin, EGF: Epidermal growth factor, OPG: Osteoprotegerin, SRC: Proto-oncogene tyrosine-protein kinase Src, IL-1ra: Interleukin-1 receptor antagonist protein, IL-6: Interleukin-6, CSTB: Cystatin-B, MCP-1: Monocyte chemotactic protein 1, KLK6: Kallikrein-6, Gal-3: Galectin-3, PAR-1: Proteinase-activated receptor 1, TRAIL: Tumor Necrosis Factor-related apoptosis-inducing ligand, hK11: Kallikrein-11, TIE2: Angiopoietin-1 receptor, TF: Tissue factor, TNF-R1: Tumor necrosis factor receptor 1, PDGF subunit B: Platelet-derived growth factor subunit B, IL27-A: Interleukin-27 subunit alpha, CSF-1: Macrophage colony-stimulating factor 1, CXCL1: C-X-C motif chemokine 1, LOX-1: Lectin-like oxidized LDL receptor 1, TRAIL-R2: Tumor necrosis factor-related apoptosis-inducing ligand receptor 2, FGF-23: Fibroblast growth factor 23, SCF: Stem cell factor, IL-18: Interleukin-18, IL-6RA: Interleukin-6 receptor subunit alpha, TNF-R2: Tumor necrosis factor receptor 2, MMP-3: Matrix metalloproteinase-3, HSP 27: Heat shock 27 kDa protein, TNFSF14: Tumor necrosis factor ligand superfamily member 14, PRL: Prolactin, MPO: Myeloperoxidase, GH: Growth hormone, MMP-1: Matrix metalloproteinase-1, RETN: Resistin, FAS: Tumor necrosis factor receptor superfamily member 6, PAPPA: Pappalysin-1, PTX3: Pentraxin-related protein, REN: Renin, CHI3L1: Chitinase-3-like protein 1, ST2:ST2 protein, TIM: TIM-1, Beta-NGF: Beta-nerve growth factor, mAmP: Membrane-bound aminopeptidase P, TRANCE: Tumor necrosis factor-related activation-induced cytokine, HGF: Hepatocyte growth factor, PSGL-1: P-selectin glycoprotein ligand 1, MB: Myoglobin, TM: Thrombomodulin, IL-16: Interleukin-16, MMP-10: Matrix metalloproteinase-10, U-PAR: Urokinase plasminogen activator surface receptor, CCL4: C-C motif chemokine 4, CTSD: Cathepsin D, RAGE: Receptor for advanced glycosylation end products, CCL3: C-C motif chemokine 3, MMP-7: Matrix metalloproteinase-7, CXCL6: C-X-C motif chemokine 16, ITGB1BP2: Melusin, CXCL16: C-X-C motif chemokine 16, Dkk-1: Dickkopf-related protein 1, SIRT2: SIR2-like protein 2, GAL: Galanin peptides, AGRP: Agouti-related protein, EN-RAGE: Protein S100-A12, CD40: Tumor necrosis factor receptor superfamily member 5, t-PA: Tissue-type plasminogen activator, HB-EGF: Heparin-binding EGF-like growth factor, ESM-1: Endothelial cell-specific molecule 1, IL-4: Interleukin-4, VEGF-D: Vascular endothelial growth factor D, MMP-12: Matrix metalloproteinase-12, SPON1: Spondin-1, CASP-8: Caspase-8, CTSL1: Cathepsin L1, CX3CL1: Fractalkine, FABP4: Fatty acid-binding protein, adipocyte, BNP: Natriuretic peptides B, LEP: Leptin, CCL20: C-C motif chemokine 20, CA-125: Ovarian cancer-related tumor marker CA 125, NEMO: NF-kappa-B essential modulator, FS: Follistatin, PECAM-1: Platelet endothelial cell adhesion molecule, NT-pro-BNP: N-terminal pro-B-type natriuretic peptide, ECP: Eosinophil cationic protein

Table S2. Proteomic markers and their association with specific sleep duration quintiles when compared to referent sleep duration (quintile 3) using 10-fold cross-fit partialing out lasso logistic regression analyses

| Sleep duration quintile | Proteomic marker | Beta coefficient | SE | z | P-value | 95% confidence interval | |
| --- | --- | --- | --- | --- | --- | --- | --- |
| Quintile 1 vs Quintile 3 | TRANCE | -0.199 | 0.084 | -2.37 | 0.018 | -0.363 – -0.035 |  |
|  | MMP-7 | 0.193 | 0.082 | 2.37 | 0.018 | 0.033 – 0.353 |  |
|  | MMP-10 | -0.160 | 0.069 | -2.32 | 0.020 | -0.295 – -0.025 |  |
|  | FS | 0.206 | 0.091 | 2.25 | 0.024 | 0.027 – 0.385 |  |
|  | SELE | 0.201 | 0.091 | 2.22 | 0.027 | 0.023 – 0.378 |  |
|  | TRAIL-R2 | 0.266 | 0.133 | 2.00 | 0.046 | 0.005 – 0.528 |  |
|  | HB-EGF | -0.226 | 0.138 | -1.64 | 0.100 | -0.498 – 0.045 |  |
|  | FAS | -0.210 | 0.135 | -1.55 | 0.120 | -0.475 – 0.056 |  |
|  | OPG | -0.175 | 0.123 | -1.43 | 0.150 | -0.415 – 0.065 |  |
|  | SCF | 0.121 | 0.087 | 1.4 | 0.160 | -0.048 – 0.291 |  |
|  | CHI3L1 | -0.113 | 0.080 | -1.41 | 0.160 | -0.269 – 0.044 |  |
|  | RAGE | -0.119 | 0.087 | -1.37 | 0.170 | -0.290 – 0.052 |  |
|  | CXCL1 | 0.113 | 0.086 | 1.32 | 0.190 | -0.055 – 0.281 |  |
|  | MB | 0.106 | 0.083 | 1.28 | 0.200 | -0.057 – 0.269 |  |
|  | VEGF-A | 0.149 | 0.118 | 1.26 | 0.210 | -0.083 – 0.381 |  |
|  | KLK6 | -0.125 | 0.102 | -1.23 | 0.220 | -0.324 – 0.075 |  |
|  | Gal-3 | -0.130 | 0.105 | -1.23 | 0.220 | -0.336 – 0.077 |  |
|  | IL-16 | -0.114 | 0.094 | -1.21 | 0.230 | -0.299 – 0.071 |  |
|  | CASP-8 | 0.107 | 0.093 | 1.16 | 0.250 | -0.074 – 0.289 |  |
|  | CX3CL1 | -0.108 | 0.094 | -1.14 | 0.250 | -0.292 – 0.077 |  |
|  | U-PAR | -0.143 | 0.127 | -1.13 | 0.260 | -0.393 – 0.106 |  |
|  | SIRT2 | -0.176 | 0.160 | -1.1 | 0.270 | -0.491 – 0.138 |  |
|  | PDGF subunit B | 0.178 | 0.168 | 1.06 | 0.290 | -0.152 – 0.508 |  |
|  | TM | 0.154 | 0.146 | 1.06 | 0.290 | -0.131 – 0.439 |  |
|  | EGF | -0.167 | 0.161 | -1.04 | 0.300 | -0.482 – 0.148 |  |
| Table S2 continued |  |  |  |  |  |  |  |
|  | AGRP | 0.097 | 0.096 | 1.01 | 0.310 | -0.091 – 0.285 |  |
|  | IL27-A | 0.088 | 0.088 | 1 | 0.320 | -0.084 – 0.261 |  |
|  | MPO | 0.090 | 0.090 | 0.99 | 0.320 | -0.088 – 0.267 |  |
|  | ESM-1 | 0.096 | 0.097 | 0.99 | 0.320 | -0.093 – 0.285 |  |
|  | CD40-L | 0.127 | 0.131 | 0.97 | 0.330 | -0.129 – 0.383 |  |
|  | HK11 | 0.095 | 0.100 | 0.96 | 0.340 | -0.100 – 0.291 |  |
|  | CD40 | 0.120 | 0.127 | 0.95 | 0.340 | -0.128 – 0.368 |  |
|  | IL-6RA | -0.082 | 0.088 | -0.94 | 0.350 | -0.254 – 0.090 |  |
|  | CSF-1 | -0.127 | 0.155 | -0.82 | 0.410 | -0.430 – 0.176 |  |
|  | CXCL6 | -0.078 | 0.095 | -0.83 | 0.410 | -0.264 – 0.107 |  |
|  | TNF-R1 | 0.169 | 0.207 | 0.81 | 0.420 | -0.237 – 0.575 |  |
|  | HSP 27 | -0.092 | 0.113 | -0.81 | 0.420 | -0.314 – 0.130 |  |
|  | PRL | -0.050 | 0.067 | -0.75 | 0.450 | -0.181 – 0.080 |  |
|  | LEP | 0.072 | 0.097 | 0.75 | 0.450 | -0.117 – 0.262 |  |
|  | NEMO | 0.089 | 0.118 | 0.75 | 0.450 | -0.142 – 0.320 |  |
|  | MCP-1 | -0.082 | 0.113 | -0.73 | 0.470 | -0.302 – 0.139 |  |
|  | IL-6 | 0.058 | 0.085 | 0.68 | 0.500 | -0.109 – 0.225 |  |
|  | PECAM-1 | -0.103 | 0.154 | -0.67 | 0.500 | -0.404 – 0.199 |  |
|  | IL-8 | 0.065 | 0.103 | 0.63 | 0.530 | -0.137 – 0.267 |  |
|  | TIE2 | 0.074 | 0.126 | 0.59 | 0.560 | -0.173 – 0.321 |  |
|  | MMP-1 | -0.041 | 0.073 | -0.57 | 0.570 | -0.185 – 0.102 |  |
|  | TRAIL | 0.057 | 0.109 | 0.52 | 0.600 | -0.156 – 0.270 |  |
|  | TIM | 0.039 | 0.076 | 0.52 | 0.610 | -0.109 – 0.188 |  |
|  | GAL | -0.039 | 0.079 | -0.5 | 0.620 | -0.193 – 0.115 |  |
|  | T-PA | -0.044 | 0.091 | -0.49 | 0.630 | -0.222 – 0.134 |  |
|  | MMP-12 | -0.039 | 0.083 | -0.47 | 0.640 | -0.200 – 0.123 |  |
|  | CCL20 | -0.034 | 0.074 | -0.47 | 0.640 | -0.179 – 0.110 |  |
| Table S2 continued |  |  |  |  |  |  |  |
|  | LOX-1 | 0.047 | 0.102 | 0.46 | 0.650 | -0.153 – 0.246 |  |
|  | AM | 0.048 | 0.116 | 0.42 | 0.680 | -0.178 – 0.275 |  |
|  | CCL3 | -0.048 | 0.116 | -0.42 | 0.680 | -0.276 – 0.179 |  |
|  | Dkk-1 | -0.067 | 0.163 | -0.41 | 0.680 | -0.386 – 0.252 |  |
|  | TF | 0.034 | 0.106 | 0.32 | 0.750 | -0.174 – 0.242 |  |
|  | RETN | 0.025 | 0.084 | 0.29 | 0.770 | -0.140 – 0.189 |  |
|  | IL-18 | 0.021 | 0.082 | 0.26 | 0.800 | -0.139 – 0.182 |  |
|  | CCL4 | -0.023 | 0.089 | -0.26 | 0.800 | -0.198 – 0.152 |  |
|  | CSTB | 0.027 | 0.112 | 0.24 | 0.810 | -0.193 – 0.246 |  |
|  | CXCL16 | 0.024 | 0.099 | 0.24 | 0.810 | -0.169 – 0.218 |  |
|  | SPON1 | 0.030 | 0.124 | 0.24 | 0.810 | -0.214 – 0.273 |  |
|  | SRC | -0.020 | 0.088 | -0.23 | 0.820 | -0.193 – 0.152 |  |
|  | CTSL1 | 0.020 | 0.096 | 0.21 | 0.840 | -0.168 – 0.207 |  |
|  | GDF-15 | 0.021 | 0.120 | 0.18 | 0.860 | -0.213 – 0.256 |  |
|  | PlGF | -0.028 | 0.162 | -0.17 | 0.860 | -0.345 – 0.290 |  |
|  | HGF | -0.024 | 0.139 | -0.17 | 0.860 | -0.296 – 0.248 |  |
|  | GH | 0.011 | 0.074 | 0.15 | 0.880 | -0.135 –0.157 |  |
|  | ECP | -0.012 | 0.089 | -0.13 | 0.890 | -0.186 – 0.162 |  |
|  | REN | 0.008 | 0.071 | 0.11 | 0.910 | -0.132 – 0.148 |  |
|  | FGF-23 | -0.008 | 0.086 | -0.09 | 0.920 | -0.177 – 0.160 |  |
|  | TNF-R2 | 0.016 | 0.175 | 0.09 | 0.930 | -0.328 –0.360 |  |
|  | CTSD | 0.008 | 0.115 | 0.07 | 0.940 | -0.218 – 0.234 |  |
|  | PAR-1 | 0.007 | 0.143 | 0.05 | 0.960 | -0.274 –0.288 |  |
|  | TNFSF14 | -0.004 | 0.115 | -0.03 | 0.970 | -0.229 – 0.222 |  |
|  | ST2 | -0.003 | 0.074 | -0.04 | 0.970 | -0.149 – 0.142 |  |
|  | VEGF-D | -0.002 | 0.082 | -0.03 | 0.980 | -0.164 –0.159 |  |
|  |  |  |  |  |  |  |  |
| Table S2 continued |  |  |  |  |  |  |  |
| Quintile 2 vs Quintile 3 | TRAIL-R2 | 0.431 | 0.143 | 3.02 | 0.0026 | 0.151 – 0.711 |  |
|  | FAS | -0.457 | 0.156 | -2.92 | 0.0035 | -0.763 – -0.150 |  |
|  | KLK6 | -0.284 | 0.111 | -2.57 | 0.010 | -0.500 – -0.067 |  |
|  | U-PAR | -0.302 | 0.140 | -2.16 | 0.031 | -0.576 – -0.0280 |  |
|  | TM | 0.298 | 0.158 | 1.890 | 0.059 | -0.011 – 0.608 |  |
|  | CTSD | -0.235 | 0.127 | -1.850 | 0.064 | -0.484 – 0.014 |  |
|  | CSTB | 0.225 | 0.123 | 1.830 | 0.067 | -0.016 – 0.465 |  |
|  | ST2 | -0.145 | 0.083 | -1.750 | 0.080 | -0.306 – 0.017 |  |
|  | Gal-3 | -0.185 | 0.110 | -1.690 | 0.092 | -0.401 – 0.030 |  |
|  | CXCL1 | 0.146 | 0.088 | 1.650 | 0.099 | -0.027 – 0.318 |  |
|  | REN | 0.115 | 0.072 | 1.590 | 0.110 | -0.026 – 0.256 |  |
|  | PECAM-1 | 0.275 | 0.170 | 1.620 | 0.110 | -0.058 – 0.607 |  |
|  | CX3CL1 | -0.162 | 0.104 | -1.560 | 0.120 | -0.365 – 0.041 |  |
|  | MPO | 0.147 | 0.102 | 1.440 | 0.150 | -0.054 – 0.348 |  |
|  | HSP 27 | -0.168 | 0.120 | -1.400 | 0.160 | -0.403 – 0.068 |  |
|  | AM | 0.181 | 0.138 | 1.310 | 0.190 | -0.090 – 0.452 |  |
|  | PAR-1 | 0.201 | 0.157 | 1.280 | 0.200 | -0.106 – 0.508 |  |
|  | FS | 0.133 | 0.103 | 1.280 | 0.200 | -0.070 – 0.336 |  |
|  | PlGF | -0.219 | 0.175 | -1.250 | 0.210 | -0.561 – 0.123 |  |
|  | HK11 | 0.140 | 0.111 | 1.260 | 0.210 | -0.078 – 0.358 |  |
|  | HB-EGF | -0.186 | 0.152 | -1.220 | 0.220 | -0.484 – 0.113 |  |
|  | IL-16 | -0.129 | 0.106 | -1.210 | 0.230 | -0.337 – 0.080 |  |
|  | T-PA | 0.118 | 0.099 | 1.190 | 0.230 | -0.076 – 0.312 |  |
|  | ESM-1 | 0.124 | 0.104 | 1.190 | 0.230 | -0.080 – 0.328 |  |
|  | TRANCE | -0.095 | 0.085 | -1.120 | 0.260 | -0.261 – 0.071 |  |
|  | RAGE | -0.104 | 0.093 | -1.120 | 0.260 | -0.287 – 0.078 |  |
|  | VEGF-A | 0.126 | 0.114 | 1.110 | 0.270 | -0.097 – 0.349 |  |
| Table S2 continued |  |  |  |  |  |  |  |
|  | VEGF-D | -0.098 | 0.089 | -1.110 | 0.270 | -0.272 – 0.076 |  |
|  | CCL20 | -0.089 | 0.083 | -1.080 | 0.280 | -0.251 – 0.073 |  |
|  | OPG | 0.138 | 0.134 | 1.030 | 0.300 | -0.125 – 0.401 |  |
|  | TIM | 0.085 | 0.084 | 1.020 | 0.310 | -0.078 – 0.249 |  |
|  | LOX-1 | -0.109 | 0.114 | -0.950 | 0.340 | -0.332 – 0114 |  |
|  | SRC | 0.073 | 0.094 | 0.780 | 0.430 | -0.111 – 0.257 |  |
|  | HGF | 0.119 | 0.150 | 0.790 | 0.430 | -0.175 – 0.413 |  |
|  | NEMO | -0.103 | 0.131 | -0.790 | 0.430 | -0.359 – 0.153 |  |
|  | CHI3L1 | 0.068 | 0.088 | 0.770 | 0.440 | -0.104 – 0.241 |  |
|  | IL-18 | 0.066 | 0.090 | 0.730 | 0.460 | -0.111 – 0.243 |  |
|  | CSF-1 | -0.121 | 0.166 | -0.730 | 0.470 | -0.446 – 0.204 |  |
|  | MMP-12 | 0.064 | 0.088 | 0.720 | 0.470 | -0.109 – 0.236 |  |
|  | CASP-8 | 0.069 | 0.099 | 0.700 | 0.480 | -0.124 – 0.262 |  |
|  | TNFSF14 | -0.083 | 0.123 | -0.680 | 0.500 | -0.324 – 0.158 |  |
|  | MMP-7 | 0.058 | 0.086 | 0.670 | 0.500 | -0.111 – 0.227 |  |
|  | CD40 | -0.096 | 0.143 | -0.680 | 0.500 | -0.376 – 0.183 |  |
|  | CTSL1 | -0.072 | 0.107 | -0.670 | 0.500 | -0.283 – 0.139 |  |
|  | TNF-R2 | 0.135 | 0.202 | 0.670 | 0.510 | -0.262 – 0.532 |  |
|  | SPON1 | 0.088 | 0.137 | 0.640 | 0.520 | -0.180 – 0.356 |  |
|  | IL-6RA | 0.055 | 0.092 | 0.600 | 0.550 | -0.126 – 0.235 |  |
|  | IL27-A | 0.053 | 0.094 | 0.560 | 0.570 | -0.132 – 0.237 |  |
|  | AGRP | 0.058 | 0.104 | 0.550 | 0.580 | -0.146 – 0.262 |  |
|  | LEP | 0.058 | 0.109 | 0.530 | 0.590 | -0.155 – 0.271 |  |
|  | IL-6 | 0.049 | 0.093 | 0.520 | 0.600 | -0.134 – 0.232 |  |
|  | GAL | 0.043 | 0.082 | 0.520 | 0.600 | -0.117 – 0.203 |  |
|  | MMP-1 | 0.039 | 0.079 | 0.490 | 0.620 | -0.117 – 0.195 |  |
|  | MCP-1 | 0.056 | 0.114 | 0.490 | 0.630 | -0.169 – 0.280 |  |
| Table S2 continued |  |  |  |  |  |  |  |
|  | TIE2 | -0.058 | 0.139 | -0.420 | 0.680 | -0.329 – 0.214 |  |
|  | TNF-R1 | -0.092 | 0.224 | -0.410 | 0.680 | -0.530 – 0.347 |  |
|  | PDGF subunit B | -0.080 | 0.195 | -0.410 | 0.680 | -0.462 –0.303 |  |
|  | MB | -0.037 | 0.090 | -0.410 | 0.680 | -0.214 –0.140 |  |
|  | CD40-L | -0.059 | 0.147 | -0.400 | 0.690 | -0.347 – 0.229 |  |
|  | GH | 0.031 | 0.077 | 0.400 | 0.690 | -0.121 – 0.183 |  |
|  | PRL | 0.028 | 0.073 | 0.380 | 0.700 | -0.115 – 0.171 |  |
|  | FGF-23 | -0.035 | 0.094 | -0.370 | 0.710 | -0.220 – 0.150 |  |
|  | IL-8 | 0.040 | 0.112 | 0.350 | 0.720 | -0.180 – 0.259 |  |
|  | TRAIL | 0.041 | 0.116 | 0.350 | 0.720 | -0.187 – 0.269 |  |
|  | CCL4 | 0.031 | 0.100 | 0.310 | 0.760 | -0.165 – 0.227 |  |
|  | MMP-10 | -0.022 | 0.075 | -0.300 | 0.770 | -0.170 – 0.125 |  |
|  | CCL3 | -0.037 | 0.124 | -0.300 | 0.770 | -0.281 – 0.207 |  |
|  | SCF | 0.023 | 0.088 | 0.260 | 0.800 | -0.150 – 0.195 |  |
|  | TF | -0.025 | 0.115 | -0.210 | 0.830 | -0.251 – 0.201 |  |
|  | ECP | 0.019 | 0.096 | 0.200 | 0.840 | -0.169 – 0.207 |  |
|  | SIRT2 | 0.033 | 0.183 | 0.180 | 0.860 | -0.327 – 0.392 |  |
|  | CXCL6 | -0.015 | 0.100 | -0.150 | 0.880 | -0.211 – 0.182 |  |
|  | CXCL16 | -0.016 | 0.103 | -0.160 | 0.880 | -0.218 – 0.186 |  |
|  | GDF-15 | -0.011 | 0.127 | -0.080 | 0.930 | -0.260 – 0.239 |  |
|  | RETN | -0.006 | 0.089 | -0.070 | 0.950 | -0.180 – 0.168 |  |
|  | Dkk-1 | 0.006 | 0.177 | 0.030 | 0.970 | -0.342 – 0.354 |  |
|  | SELE | 0.001 | 0.099 | 0.010 | 0.990 | -0.193 – 0.195 |  |
|  | EGF | 0.002 | 0.179 | 0.010 | 0.990 | -0.349 –0.352 |  |
|  |  |  |  |  |  |  |  |
|  |  |  |  |  |  |  |  |
|  |  |  |  |  |  |  |  |
| Table S2 continued |  |  |  |  |  |  |  |
| Quintile 4 vs Quintile 3 | FAS | -0.452 | 0.135 | -3.34 | 0.00084 | -0.717 – -0.187 |  |
|  | REN | 0.186 | 0.070 | 2.64 | 0.0082 | 0.048 – 0.324 |  |
|  | HB-EGF | -0.329 | 0.135 | -2.44 | 0.015 | -0.594 – -0.064 |  |
|  | MMP-7 | 0.189 | 0.081 | 2.34 | 0.019 | 0.031 – 0.348 |  |
|  | CXCL6 | 0.210 | 0.097 | 2.16 | 0.031 | 0.019 – 0.400 |  |
|  | CXCL1 | 0.150 | 0.081 | 1.850 | 0.064 | -0.009 – 0.309 |  |
|  | RETN | 0.156 | 0.086 | 1.800 | 0.071 | -0.013 – 0.325 |  |
|  | IL-16 | -0.182 | 0.101 | -1.800 | 0.071 | -0.381 – 0.016 |  |
|  | KLK6 | -0.154 | 0.100 | -1.540 | 0.120 | -0.350 – 0.042 |  |
|  | SPON1 | -0.198 | 0.127 | -1.560 | 0.120 | -0.448 – 0.051 |  |
|  | T-PA | 0.139 | 0.094 | 1.470 | 0.140 | -0.046 –0.323 |  |
|  | HSP 27 | -0.163 | 0.112 | -1.460 | 0.150 | -0.383 – 0.056 |  |
|  | CHI3L1 | 0.098 | 0.078 | 1.250 | 0.210 | -0.055 – 0.250 |  |
|  | U-PAR | -0.169 | 0.133 | -1.270 | 0.210 | -0.430 – 0.092 |  |
|  | ESM-1 | 0.119 | 0.096 | 1.230 | 0.220 | -0.070 – 0.308 |  |
|  | MMP-12 | 0.104 | 0.086 | 1.210 | 0.230 | -0.065 – 0.273 |  |
|  | PDGF subunit B | 0.212 | 0.180 | 1.180 | 0.240 | -0.141 – 0.566 |  |
|  | PRL | 0.081 | 0.071 | 1.140 | 0.250 | -0.058 – 0.220 |  |
|  | TF | 0.123 | 0.111 | 1.110 | 0.270 | -0.095 – 0.342 |  |
|  | MPO | 0.102 | 0.096 | 1.060 | 0.290 | -0.086 – 0.289 |  |
|  | CCL4 | -0.090 | 0.088 | -1.020 | 0.310 | -0.262 – 0.083 |  |
|  | AGRP | 0.094 | 0.093 | 1.010 | 0.310 | -0.088 – 0.276 |  |
|  | AM | -0.114 | 0.119 | -0.950 | 0.340 | -0.347 – 0.120 |  |
|  | SRC | 0.085 | 0.088 | 0.960 | 0.340 | -0.088 – 0.257 |  |
|  | PAR-1 | 0.137 | 0.145 | 0.950 | 0.340 | -0.146 – 0.420 |  |
|  | CCL3 | 0.111 | 0.116 | 0.960 | 0.340 | -0.115 – 0.338 |  |
|  | Dkk-1 | -0.149 | 0.160 | -0.930 | 0.350 | -0.462 – 0.164 |  |
| Table S2 continued |  |  |  |  |  |  |  |
|  | CSTB | 0.096 | 0.116 | 0.820 | 0.410 | -0.133 – 0.324 |  |
|  | TNF-R1 | 0.157 | 0.204 | 0.770 | 0.440 | -0.243 – 0.556 |  |
|  | IL-8 | -0.079 | 0.110 | -0.720 | 0.470 | -0.294 – 0.136 |  |
|  | CSF-1 | -0.117 | 0.160 | -0.730 | 0.470 | -0.430 – 0.197 |  |
|  | GDF-15 | 0.083 | 0.118 | 0.710 | 0.480 | -0.147 – 0.314 |  |
|  | CX3CL1 | 0.069 | 0.098 | 0.700 | 0.480 | -0.123 – 0.260 |  |
|  | OPG | 0.079 | 0.122 | 0.650 | 0.510 | -0.160 – 0.319 |  |
|  | TRAIL-R2 | 0.090 | 0.135 | 0.670 | 0.510 | -0.175 – 0.355 |  |
|  | TRANCE | -0.052 | 0.081 | -0.640 | 0.520 | -0.212 – 0.107 |  |
|  | MCP-1 | -0.069 | 0.110 | -0.630 | 0.530 | -0.284 – 0.146 |  |
|  | PECAM-1 | 0.098 | 0.155 | 0.630 | 0.530 | -0.206 –0.402 |  |
|  | MB | -0.052 | 0.084 | -0.610 | 0.540 | -0.217 – 0.114 |  |
|  | CASP-8 | 0.058 | 0.096 | 0.600 | 0.550 | -0.130 – 0.246 |  |
|  | TNF-R2 | 0.098 | 0.173 | 0.560 | 0.570 | -0.242 – 0.437 |  |
|  | CTSL1 | 0.053 | 0.096 | 0.550 | 0.580 | -0.135 – 0.240 |  |
|  | CTSD | -0.062 | 0.116 | -0.540 | 0.590 | -0.291 – 0.166 |  |
|  | GH | 0.040 | 0.076 | 0.530 | 0.600 | -0.109 – 0.189 |  |
|  | EGF | -0.083 | 0.164 | -0.510 | 0.610 | -0.404 – 0.238 |  |
|  | GAL | 0.040 | 0.079 | 0.500 | 0.610 | -0.115 – 0.195 |  |
|  | TRAIL | -0.055 | 0.110 | -0.500 | 0.620 | -0.270 – 0.161 |  |
|  | MMP-10 | -0.035 | 0.070 | -0.490 | 0.620 | -0.172 – 0.103 |  |
|  | RAGE | 0.044 | 0.089 | 0.490 | 0.620 | -0.131 – 0.218 |  |
|  | LEP | 0.046 | 0.094 | 0.490 | 0.620 | -0.137 – 0.230 |  |
|  | IL-6 | 0.040 | 0.085 | 0.470 | 0.640 | -0.126 – 0.206 |  |
|  | IL-6RA | -0.040 | 0.089 | -0.450 | 0.650 | -0.214 – 0.134 |  |
|  | FS | 0.039 | 0.095 | 0.410 | 0.680 | -0.148 – 0.225 |  |
|  | SCF | 0.034 | 0.084 | 0.400 | 0.690 | -0.131 – 0.198 |  |
| Table S2 continued |  |  |  |  |  |  |  |
|  | TIM | 0.028 | 0.078 | 0.360 | 0.720 | -0.124 – 0.180 |  |
|  | TM | -0.047 | 0.144 | -0.330 | 0.740 | -0.330 – 0.235 |  |
|  | SIRT2 | -0.055 | 0.164 | -0.330 | 0.740 | -0.376 – 0.267 |  |
|  | Gal-3 | -0.033 | 0.106 | -0.310 | 0.760 | -0.240 – 0.175 |  |
|  | HK11 | -0.028 | 0.097 | -0.290 | 0.770 | -0.218 – 0.162 |  |
|  | SELE | 0.025 | 0.092 | 0.280 | 0.780 | -0.155 – 0.205 |  |
|  | ECP | 0.022 | 0.089 | 0.250 | 0.800 | -0.152 – 0.196 |  |
|  | TNFSF14 | 0.024 | 0.115 | 0.210 | 0.830 | -0.201 – 0.249 |  |
|  | FGF-23 | -0.017 | 0.086 | -0.200 | 0.840 | -0.186 – 0.152 |  |
|  | LOX-1 | -0.020 | 0.104 | -0.190 | 0.850 | -0.223 – 0.184 |  |
|  | CD40-L | -0.024 | 0.133 | -0.180 | 0.860 | -0.284 – 0.236 |  |
|  | MMP-1 | 0.013 | 0.074 | 0.170 | 0.870 | -0.132 – 0.157 |  |
|  | PlGF | -0.021 | 0.162 | -0.130 | 0.900 | -0.337 – 0.296 |  |
|  | CD40 | -0.017 | 0.133 | -0.130 | 0.900 | -0.277 – 0.243 |  |
|  | CXCL16 | -0.011 | 0.098 | -0.110 | 0.910 | -0.202 – 0.181 |  |
|  | VEGF-A | -0.011 | 0.116 | -0.100 | 0.920 | -0.239 – 0.216 |  |
|  | IL-18 | -0.008 | 0.083 | -0.100 | 0.920 | -0.171 – 0.155 |  |
|  | HGF | -0.014 | 0.133 | -0.100 | 0.920 | -0.275 – 0.247 |  |
|  | TIE2 | -0.008 | 0.127 | -0.070 | 0.950 | -0.257 – 0.240 |  |
|  | VEGF-D | -0.005 | 0.089 | -0.050 | 0.960 | -0.178 – 0.169 |  |
|  | CCL20 | -0.004 | 0.077 | -0.050 | 0.960 | -0.156 – 0.148 |  |
|  | NEMO | -0.006 | 0.124 | -0.050 | 0.960 | -0.248 – 0.237 |  |
|  | ST2 | -0.002 | 0.077 | -0.030 | 0.980 | -0.153 – 0.149 |  |
|  | IL27-A | 0.001 | 0.087 | 0.020 | 0.990 | -0.169 – 0.171 |  |
|  |  |  |  |  |  |  |  |
|  |  |  |  |  |  |  |  |
|  |  |  |  |  |  |  |  |
| Table S2 continued |  |  |  |  |  |  |  |
| Quintile 5 vs Quintile 3 | PRL | 0.261 | 0.081 | 3.22 | 0.0013 | 0.102 – 0.420 |  |
|  | MMP-7 | 0.296 | 0.097 | 3.05 | 0.0023 | 0.106 – 0.486 |  |
|  | CXCL1 | 0.245 | 0.100 | 2.44 | 0.015 | 0.0483 – 0.441 |  |
|  | FAS | -0.402 | 0.170 | -2.37 | 0.018 | -0.735 – -0.070 |  |
|  | t-PA | 0.256 | 0.110 | 2.32 | 0.020 | 0.0395 – 0.472 |  |
|  | HSP27 | -0.284 | 0.130 | -2.19 | 0.029 | -0.538 – -0.030 |  |
|  | IL-16 | -0.226 | 0.118 | -1.910 | 0.056 | -0.458 – 0.006 |  |
|  | CTSD | 0.250 | 0.131 | 1.910 | 0.056 | -0.007 – 0.508 |  |
|  | REN | 0.149 | 0.083 | 1.800 | 0.072 | -0.013 – 0.311 |  |
|  | IL-8 | 0.215 | 0.126 | 1.710 | 0.087 | -0.031 – 0.461 |  |
|  | ST2 | -0.150 | 0.090 | -1.660 | 0.096 | -0.326 – 0.027 |  |
|  | CCL20 | -0.149 | 0.090 | -1.650 | 0.098 | -0.326 – 0.028 |  |
|  | EGF | -0.307 | 0.194 | -1.580 | 0.110 | -0.688 – 0.074 |  |
|  | TNF-R1 | 0.408 | 0.253 | 1.610 | 0.110 | -0.088 – 0.904 |  |
|  | MB | -0.145 | 0.099 | -1.470 | 0.140 | -0.339 – 0.049 |  |
|  | CXCL6 | -0.164 | 0.119 | -1.380 | 0.170 | -0.398 – 0.070 |  |
|  | TF | -0.178 | 0.136 | -1.310 | 0.190 | -0.444 – 0.088 |  |
|  | MPO | 0.138 | 0.111 | 1.250 | 0.210 | -0.079 – 0.356 |  |
|  | Gal-3 | -0.153 | 0.125 | -1.230 | 0.220 | -0.398 – 0.091 |  |
|  | GAL | 0.114 | 0.094 | 1.210 | 0.220 | -0.070 – 0.297 |  |
|  | TNFSF14 | 0.168 | 0.141 | 1.190 | 0.230 | -0.108 – 0.445 |  |
|  | MMP-1 | 0.098 | 0.087 | 1.120 | 0.260 | -0.073 – 0.269 |  |
|  | TM | 0.189 | 0.172 | 1.090 | 0.270 | -0.149 – 0.527 |  |
|  | VEGF-D | -0.113 | 0.102 | -1.110 | 0.270 | -0.314 – 0.088 |  |
|  | CD40-L | 0.172 | 0.164 | 1.050 | 0.290 | -0.150 – 0.495 |  |
|  | SCF | 0.105 | 0.101 | 1.040 | 0.300 | -0.093 – 0.304 |  |
|  | CHI3L1 | -0.095 | 0.094 | -1.010 | 0.310 | -0.279 – 0.089 |  |
| Table S2 continued |  |  |  |  |  |  |  |
|  | HB-EGF | -0.144 | 0.156 | -0.930 | 0.350 | -0.449 – 0.161 |  |
|  | CTSL1 | -0.103 | 0.114 | -0.900 | 0.370 | -0.327 – 0.121 |  |
|  | TIM | -0.078 | 0.093 | -0.840 | 0.400 | -0.260 – 0.104 |  |
|  | TRAIL-R2 | -0.139 | 0.170 | -0.820 | 0.410 | -0.473 – 0.195 |  |
|  | RAGE | -0.088 | 0.107 | -0.820 | 0.410 | -0.297 – 0.122 |  |
|  | NEMO | -0.119 | 0.144 | -0.830 | 0.410 | -0.401 –0.163 |  |
|  | KLK6 | -0.092 | 0.121 | -0.760 | 0.440 | -0.329 – 0.145 |  |
|  | PAR-1 | 0.130 | 0.168 | 0.770 | 0.440 | -0.200 – 0.460 |  |
|  | MCP-1 | -0.098 | 0.138 | -0.710 | 0.470 | -0.368 – 0.171 |  |
|  | FGF-23 | 0.069 | 0.101 | 0.680 | 0.490 | -0.129 – 0.267 |  |
|  | IL-6 | 0.070 | 0.104 | 0.670 | 0.500 | -0.134 – 0.274 |  |
|  | PDGF subunit B | -0.137 | 0.201 | -0.680 | 0.500 | -0.531 – 0.258 |  |
|  | TRAIL | -0.082 | 0.127 | -0.650 | 0.520 | -0.331 – 0.167 |  |
|  | ECP | 0.064 | 0.099 | 0.650 | 0.520 | -0.130 – 0.258 |  |
|  | MMP-10 | 0.051 | 0.084 | 0.610 | 0.540 | -0.112 – 0.215 |  |
|  | CXCL16 | -0.065 | 0.112 | -0.580 | 0.560 | -0.284 –0.155 |  |
|  | HK11 | -0.067 | 0.118 | -0.560 | 0.570 | -0.298 –0.165 |  |
|  | CX3CL1 | -0.059 | 0.118 | -0.500 | 0.620 | -0.290 – 0.172 |  |
|  | FS | 0.057 | 0.116 | 0.490 | 0.620 | -0.170 – 0.284 |  |
|  | CCL4 | 0.049 | 0.107 | 0.460 | 0.650 | -0.161 – 0.259 |  |
|  | PECAM-1 | 0.084 | 0.185 | 0.450 | 0.650 | -0.279 – 0.447 |  |
|  | SELE | -0.043 | 0.108 | -0.400 | 0.690 | -0.255 – 0.169 |  |
|  | CSF-1 | -0.057 | 0.168 | -0.340 | 0.730 | -0.386 – 0.271 |  |
|  | U-PAR | -0.040 | 0.155 | -0.260 | 0.790 | -0.344 – 0.264 |  |
|  | Dkk-1 | 0.049 | 0.183 | 0.270 | 0.790 | -0.309 – 0.407 |  |
|  | IL27-A | 0.026 | 0.104 | 0.250 | 0.800 | -0.177 – 0.229 |  |
|  | VEGF-A | 0.030 | 0.131 | 0.230 | 0.820 | -0.227 – 0.288 |  |
| Table S2 continued |  |  |  |  |  |  |  |
|  | HGF | 0.035 | 0.158 | 0.220 | 0.820 | -0.275 – 0.345 |  |
|  | PlGF | 0.040 | 0.186 | 0.210 | 0.830 | -0.324 – 0.404 |  |
|  | CSTB | 0.029 | 0.137 | 0.210 | 0.830 | -0.239 – 0.297 |  |
|  | SPON1 | -0.031 | 0.146 | -0.220 | 0.830 | -0.318 – 0.255 |  |
|  | GDF-15 | 0.028 | 0.145 | 0.190 | 0.850 | -0.257 – 0.313 |  |
|  | TRANCE | -0.018 | 0.096 | -0.190 | 0.850 | -0.206 – 0.169 |  |
|  | MMP-12 | -0.018 | 0.101 | -0.170 | 0.860 | -0.215 – 0.179 |  |
|  | GH | 0.013 | 0.088 | 0.150 | 0.880 | -0.159 – 0.185 |  |
|  | RETN | 0.014 | 0.103 | 0.140 | 0.890 | -0.187 – 0.215 |  |
|  | SIRT2 | 0.027 | 0.195 | 0.140 | 0.890 | -0.355 – 0.409 |  |
|  | SRC | -0.013 | 0.107 | -0.120 | 0.900 | -0.224 – 0.197 |  |
|  | TNF-R2 | 0.027 | 0.222 | 0.120 | 0.900 | -0.409 – 0.462 |  |
|  | CCL3 | -0.016 | 0.135 | -0.120 | 0.910 | -0.281 – 0.249 |  |
|  | CD40 | -0.017 | 0.151 | -0.120 | 0.910 | -0.314 – 0.279 |  |
|  | ESM-1 | 0.010 | 0.112 | 0.080 | 0.930 | -0.211 –0.230 |  |
|  | LEP | 0.011 | 0.121 | 0.090 | 0.930 | -0.227 – 0.249 |  |
|  | AM | -0.011 | 0.140 | -0.080 | 0.940 | -0.285 – 0.263 |  |
|  | OPG | 0.011 | 0.145 | 0.080 | 0.940 | -0.273 –0.296 |  |
|  | LOX-1 | -0.008 | 0.123 | -0.070 | 0.950 | -0.250 – 0.234 |  |
|  | AGRP | 0.006 | 0.117 | 0.050 | 0.960 | -0.222 – 0.235 |  |
|  | CASP-8 | 0.006 | 0.112 | 0.050 | 0.960 | -0.213 – 0.225 |  |
|  | IL-18 | 0.003 | 0.097 | 0.030 | 0.980 | -0.187 – 0.192 |  |
|  | IL-6RA | -0.003 | 0.107 | -0.030 | 0.980 | -0.212 – 0.206 |  |
|  | TIE2 | 0.002 | 0.146 | 0.010 | 0.990 | -0.283 – 0.287 |  |
|  |  |  |  |  |  |  |  |

Abbreviations: AGRP: Agouti-related protein, AM: Adrenomedullin, CASP-8: Caspase-8, CCL3: C-C motif chemokine 3, CCL4: C-C motif chemokine 4, CCL20: C-C motif chemokine 20, CD40: Tumor necrosis factor receptor superfamily member 5, CD40-L: CD40 ligand, CHI3L1: Chitinase-3-like protein 1, CSF-1: Macrophage colony-stimulating factor 1, CSTB: Cystatin-B, CTSD: Cathepsin D, CTSL1: Cathepsin L1, CX3CL1: Fractalkine, CXCL1: C-X-C motif chemokine 1, CXCL6: C-X-C motif chemokine 6, CXCL16: C-X-C motif chemokine 16, Dkk-1: Dickkopf-related protein 1, ECP: Eosinophil cationic protein, EGF: Epidermal growth factor, ESM-1: Endothelial cell-specific molecule 1, FAS: Tumor necrosis factor receptor superfamily member 6, FGF-23: Fibroblast growth factor 23, FS: Follistatin, GAL: Galanin peptides, Gal-3: Galectin-3, GDF-15: Growth/differentiation factor 15, GH: Growth hormone, HB-EGF: Heparin-binding EGF-like growth factor, HGF: Hepatocyte growth factor, hK11: Kallikrein-11, HSP 27: Heat shock 27 kDa protein, IL-6: Interleukin-6, IL-6RA: Interleukin-6 receptor subunit alpha, IL-8: Interleukin-8, IL-16: Interleukin-16, IL-18: Interleukin-18, IL27-A: Interleukin-27 subunit alpha, KLK6: Kallikrein-6, LEP: Leptin, LOX-1: Lectin-like oxidized LDL receptor 1, MB: Myoglobin, MCP-1: Monocyte chemotactic protein 1, MMP-1: Matrix metalloproteinase-1, MMP-7: Matrix metalloproteinase-7, MMP-10: Matrix metalloproteinase-10, MMP-12: Matrix metalloproteinase-12, MPO: Myeloperoxidase, NEMO: NF-kappa-B essential modulator, OPG: Osteoprotegerin, PlGF: Placenta growth factor, PAR-1: Proteinase-activated receptor 1, PDGF subunit B: Platelet-derived growth factor subunit B, PECAM-1: Platelet endothelial cell adhesion molecule, PRL: Prolactin, RAGE: Receptor for advanced glycosylation end products, REN: Renin, RETN: Resistin, SCF: Stem cell factor, SELE: E-selectin, SIRT2: SIR2-like protein 2, SPON1: Spondin-1, SRC: Proto-oncogene tyrosine-protein kinase Src, ST2:ST2 protein, TF: Tissue factor, TIE2: Angiopoietin-1 receptor, TIM: TIM-1, TM: Thrombomodulin, TNF-R1: Tumor necrosis factor receptor 1, TNF-R2: Tumor necrosis factor receptor 2, TNFSF14: Tumor necrosis factor ligand superfamily member 14, t-PA: Tissue-type plasminogen activator, TRAIL: Tumor Necrosis Factor-related apoptosis-inducing ligand, TRAIL-R2: Tumor necrosis factor-related apoptosis-inducing ligand receptor 2, TRANCE: Tumor necrosis factor-related activation-induced cytokine, U-PAR: Urokinase plasminogen activator surface receptor, VEGF-A: Vascular endothelial growth factor A, VEGF-D: Vascular endothelial growth factor D
